# Tinnitus-like “hallucinations” elicited by sensory deprivation in an entropy maximization recurrent neural network

**DOI:** 10.1371/journal.pcbi.1008664

**Published:** 2021-12-08

**Authors:** Aviv Dotan, Oren Shriki

**Affiliations:** 1 Department of Cognitive and Brain Sciences, Ben-Gurion University of the Negev, Beer-Sheva, Israel; 2 Department of Computer Science, Ben-Gurion University of the Negev, Beer-Sheva, Israel; 3 Zlotowski Center for Neuroscience, Ben-Gurion University of the Negev, Beer-Sheva, Israel; University College London, UNITED KINGDOM

## Abstract

Sensory deprivation has long been known to cause hallucinations or “phantom” sensations, the most common of which is tinnitus induced by hearing loss, affecting 10–20% of the population. An observable hearing loss, causing auditory sensory deprivation over a band of frequencies, is present in over 90% of people with tinnitus. Existing plasticity-based computational models for tinnitus are usually driven by homeostatic mechanisms, modeled to fit phenomenological findings. Here, we use an objective-driven learning algorithm to model an early auditory processing neuronal network, e.g., in the dorsal cochlear nucleus. The learning algorithm maximizes the network’s output entropy by learning the feed-forward and recurrent interactions in the model. We show that the connectivity patterns and responses learned by the model display several hallmarks of early auditory neuronal networks. We further demonstrate that attenuation of peripheral inputs drives the recurrent network towards its critical point and transition into a tinnitus-like state. In this state, the network activity resembles responses to genuine inputs even in the absence of external stimulation, namely, it “hallucinates” auditory responses. These findings demonstrate how objective-driven plasticity mechanisms that normally act to optimize the network’s input representation can also elicit pathologies such as tinnitus as a result of sensory deprivation.

## Author summary

Tinnitus or “ringing in the ears” is a common pathology. It may result from mechanical damage in the inner ear, as well as from certain drugs such as salicylate (aspirin). A common approach toward a computational model for tinnitus is to use a neural network model with inherent plasticity applied to early auditory processing, where the input layer models the auditory nerve and the output layer models a nucleus in the brain stem. However, most of the existing computational models are phenomenological in nature, driven by a homeostatic principle. Here, we use an objective-driven learning algorithm based on information theory to learn the feed-forward interactions between the layers, as well as the recurrent interactions within the output layer. Through numerical simulations of the learning process, we show that attenuation of peripheral inputs drives the network into a tinnitus-like state, where the network activity resembles responses to genuine inputs even in the absence of external stimulation; namely, it “hallucinates” auditory responses. These findings demonstrate how plasticity mechanisms that normally act to optimize network performance can also lead to undesired outcomes, such as tinnitus, as a result of reduced peripheral hearing.

## Introduction

Tinnitus is a common form of auditory hallucinations, affecting the quality of life of many people (≈ 10–20% of the population, [1–6]). It can manifest as a “ringing” or hissing sound across a certain frequency range, typically with a distinct spectral peak [7, 8]. An observable hearing loss, causing sensory deprivation over a band of frequencies, is present in >90% of people with tinnitus [1–4], and the remaining people with tinnitus are believed to suffer some damage in higher auditory processing pathways [5, 9] or have some cochlear damage that does not affect the audiogram [10].

From a neural processing point of view, hallucinations correspond to brain activity in sensory networks, which occurs in the absence of an objective external input. Hallucinations can occur in all sensory modalities, and can be induced by drugs, certain brain disorders, and sensory deprivation. For example, it is well known that visual deprivation (e.g., being in darkness for an extended period) elicits visual hallucinations, and, similarly, auditory deprivation elicits auditory hallucinations [11–13].

Although the causes of tinnitus can sometimes be mechanical (“objective tinnitus” [2, 14]), this is not the case in >95% of patients [6, 14]. This so-called “subjective tinnitus” is commonly associated with plasticity of feedback and recurrent neuronal circuits [2, 5, 10, 15–18].

The dorsal cochlear nucleus (DCN) is known to display tinnitus-related plastic reorganization following cochlear damage [19–22], and is thought to be a key player in the generation of tinnitus [23–26]. It is stimulated directly by the auditory nerve with a tonotopic mapping. Each output unit, composed of a group of different cells, receives inputs from a small number of input fibers and inhibits units of similar tuning [27, 28]. This connectivity pattern results in a sharp detection of specific notches [28]. As the DCN is the foremost anatomical structure in the auditory pathway in which tinnitus-related activity has been observed [19, 20], it is the structure most associated with the generation of tinnitus [23–26]. This choice is also supported by DCN hyperactivity following artificial induction of tinnitus [21, 22]. Interestingly, this induced hyperactivity seems to persist even if the DCN is later isolated from inputs other than the auditory nerve [29]. This suggests that tinnitus-related hyperactivity in the DCN is self-sustained and does not depend on feedback from higher order auditory networks.

The DCN also receives non-auditory inputs, such as somatosensory and vestibular projections [30–33]. The somatosensory projections, in particular, are known to be upregulated in tinnitus [22, 34–38]. Furthermore, somatosensory stimulation is known to affect the perceived tinnitus in >60% of the cases [37, 39, 40]. In light of these observations, the somatosensory projections are considered to play a major role in tinnitus [37]. A recent study used a bimodal auditory-sensory stimulation as a treatment paradigm in both guinea pigs and humans, successfully modulating the percept of tinnitus and reducing its loudness, though the effect did not last after terminating the treatment [41].

While existing computational models successfully account for some of the characteristics of tinnitus [42], many of them are based on lateral inhibition [43–45] or gain adaptation [46], and do not take into account long-term neural plasticity. Plasticity-based models for tinnitus are usually phenomenological models, where plasticity is described as a homeostatic process [47–53] or an amplification of central noise [54], and not as a process which serves a computational goal. Another computational model for tinnitus is based on stochastic resonance and suggests that tinnitus arises from an adaptive optimal noise level [55, 56]. This model successfully accounts for various aspects of tinnitus and other auditory phenomena related to sensory deprivation, but it is focused on a single auditory frequency and has yet to be further explored in a broader context.

In this work, we try to gain new insights into tinnitus by using information theoretic-driven plasticity. We implemented the entropy maximization (EM) approach in a recurrent neural network [57] to model the connection between the raw sensory input and its downstream representation. This approach was previously applied to model the feed-forward connectivity in the primary visual cortex, giving rise to orientation-selective Gabor-like receptive fields [58]. A later generalization of the algorithm to learning recurrent connectivity [57] was used to show that EM drives early visual processing networks toward critical behavior [59]. Furthermore, the evolved recurrent connectivity profile has a Mexican-hat shape; namely, neurons with similar preferred orientations tend to excite one another, while neurons with distant preferred orientations tend to inhibit one another, consistent with empirical data. While the aforementioned studies focused on the normal function of the visual system, EM-based neural networks were barely used to model abnormalities or to study the effect of changes in input statistics [60]. The relationship between EM-based adaptation and the emergence of tinnitus from sensory deprivation was previously discussed in the context of single neurons [61], yet it was never explored on a large-scale recurrent network.

Here, we trained a recurrent EM neural network to represent auditory stimuli, so it can stand as a simplified model for early auditory processing. Subsequently, to test the effect of sensory deprivation on the network’s output representation, we modified the input statistics by attenuating a certain range of frequencies. Our findings show that tinnitus-like hallucinations naturally arise in this model following sensory deprivation. Specifically, the recurrent interactions act to compensate for the attenuated input by increasing their gain, causing the network to cross a critical point into a regime of hallucinations. These findings suggest that hallucinations following sensory deprivation can stem from general long-term plasticity mechanisms that act to optimize the representation of sensory information.

## Results

**Fig 1:**
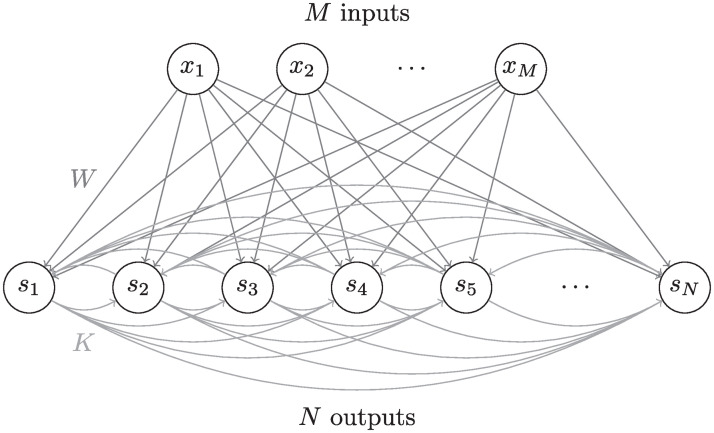
Network’s architecture. The architecture of an overcomplete recurrent neural network with *M* input neurons and *N* output neurons, where *N* > *M*.

**Fig 2:**
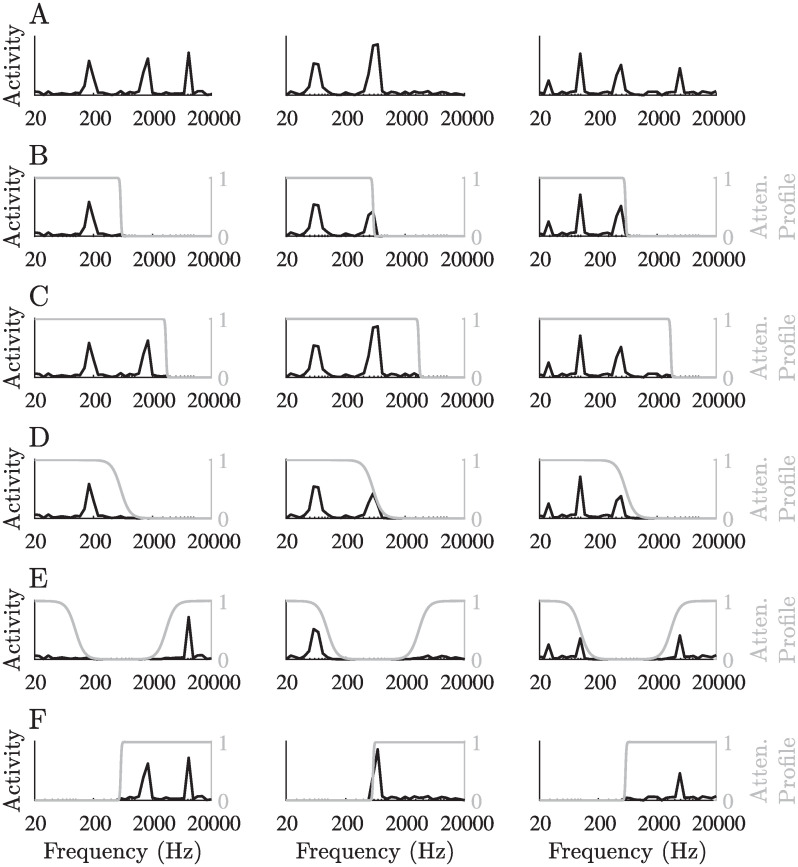
Typical stimuli. A: Three examples of typical simulated stimuli, representing the activity of the input neurons as a function of their preferred frequency. B–F: The stimuli presented in A, after attenuation of different frequency ranges using different attenuation profiles. Attenuation was achieved by multiplying the original input vector by an attenuation profile, depicted in gray. The attenuation profiles in B–D and F were inverted sigmoidal functions with parameters *k*_0_ = 20, *β* = 10 for B, *k*_0_ = 30, *β* = 10 for C, *k*_0_ = 20, *β* = 1 for D and *k*_0_ = 20, *β* = −10 for F, where *k*_0_ represents the transition frequency (in the input neurons domain, between 1 and *M* = 40) and *β* represents the sharpness of the transition. The attenuation profile in E was composed of two sigmoidal functions with parameters *k*_1_ = 10, *k*_2_ = 30, *β* = 1. For further details, see Methods.

To model the early stages of auditory processing (e.g., DCN), we used an EM approach to train a recurrent neural network (see Methods). The neurons obey first-order rate dynamics, and it is assumed that the network reaches a steady state following the presentation of each stimulus. The learning algorithm for the feed-forward and recurrent connectivity was based on the gradient-descent algorithm described in [57], with the addition of regularization. The network was trained in an unsupervised manner to represent simulated auditory stimuli (see Methods for more details). Figs 1 and 2A depict the network’s architecture and typical stimuli, respectively.

In all simulations described here, we used a network of 40 input neurons and 400 output neurons (an overcomplete representation). Regularization was achieved using a cost on the norm of the weights and was applied to both feed-forward (using *ℓ*_1_ norm) and recurrent (using *ℓ*_2_ norm) sets of connections (see Methods). The coefficients of the regularization terms were set to λ_*W*_ = 0.001 for the feed-forward connections and λ_*K*_ = 0.226 for the recurrent connections (for details regarding these choices, see below the subsection on the Regularization effect).

### Training using typical stimuli

**Fig 3:**
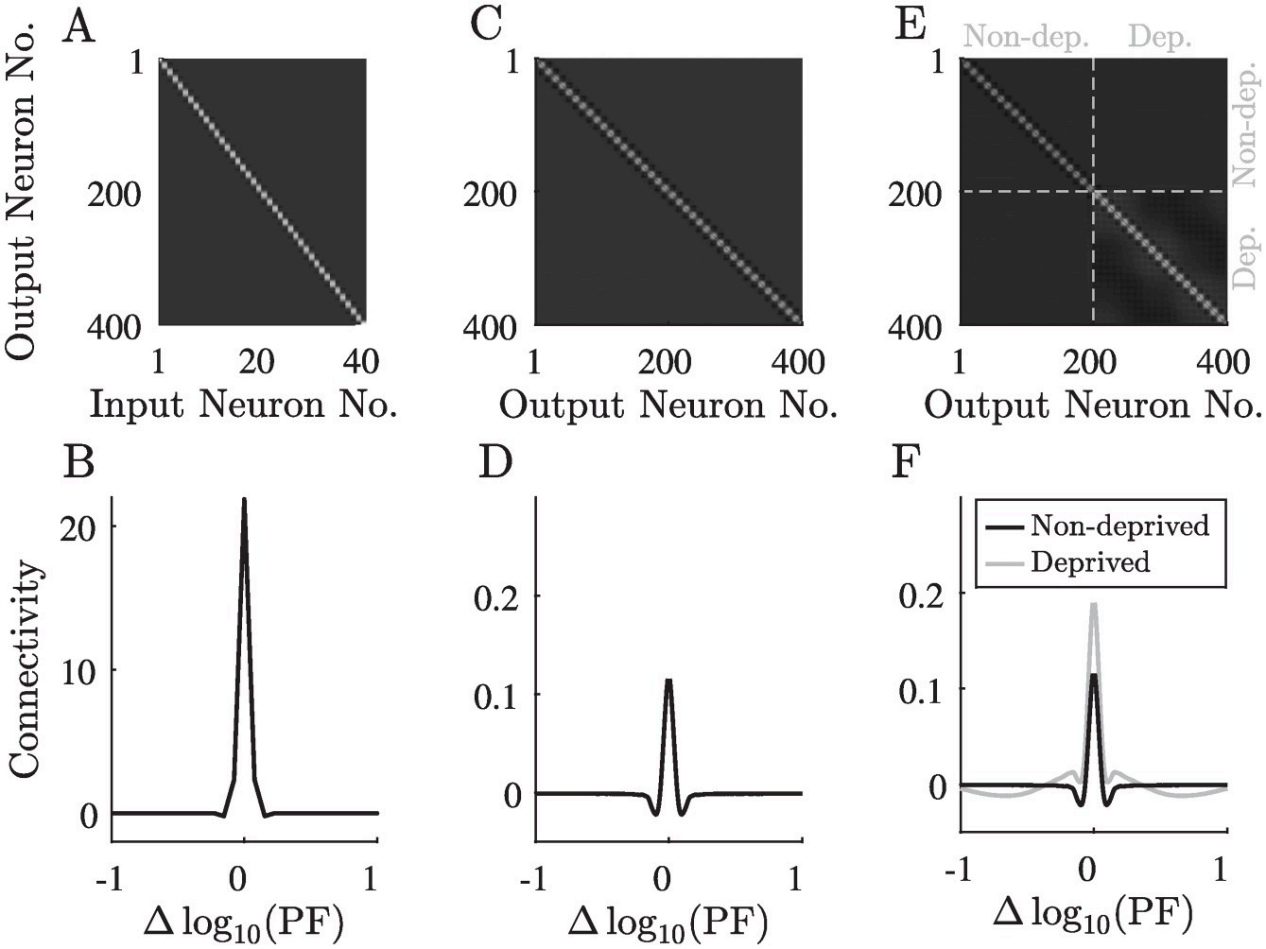
The network’s connectivity before and after sensory deprivation. A–B: The feed-forward connectivity matrix and its average row profile. C–D: The recurrent connectivity matrix and its average row profile before sensory deprivation. E–F: The recurrent connectivity matrix and its average row profiles after sensory deprivation, averaged separately for neurons in the deprived zone and the non-deprived zone. Each row profile is obtained by aligning the presynaptic connections to every neuron according to its preferred frequency and then averaging. The x-axis in B, D and F describes the log-scaled difference in the preferred frequency between the presynaptic and postsynaptic neurons. The attenuation profile’s parameters were *k*_0_ = 20, *β* = 10 (see Fig 2B). The classification of output neurons into deprived and non-deprived zones in F is based on the level of attenuation at the preferred frequency of the neuron.

First, we trained the network using typical auditory inputs, simulated as a combination of multiple narrow Gaussians in the log-scaled frequency domain with additional noise (see Methods and Fig 2A). After the convergence of the learning process, each output neuron had a specific and unique preferred frequency, as manifested in the feed-forward connectivity profiles (Fig 3A and 3B). The recurrent connections converged to a “Mexican-hat” profile with short-range excitation and longer-range inhibition (Fig 3C and 3D). This profile of connectivity causes neurons with adjacent frequencies to excite one another, while neurons with slightly more distant frequencies inhibit each other. The significance of this profile lies in its ability to reduce the width of the output response profile for a Gaussian input, thus, effectively reducing the noise. Similarly shaped spectral receptive fields were observed in various primary auditory networks [27, 28, 62, 63] including the DCN, suggesting similar connectivity patterns.

**Fig 4:**
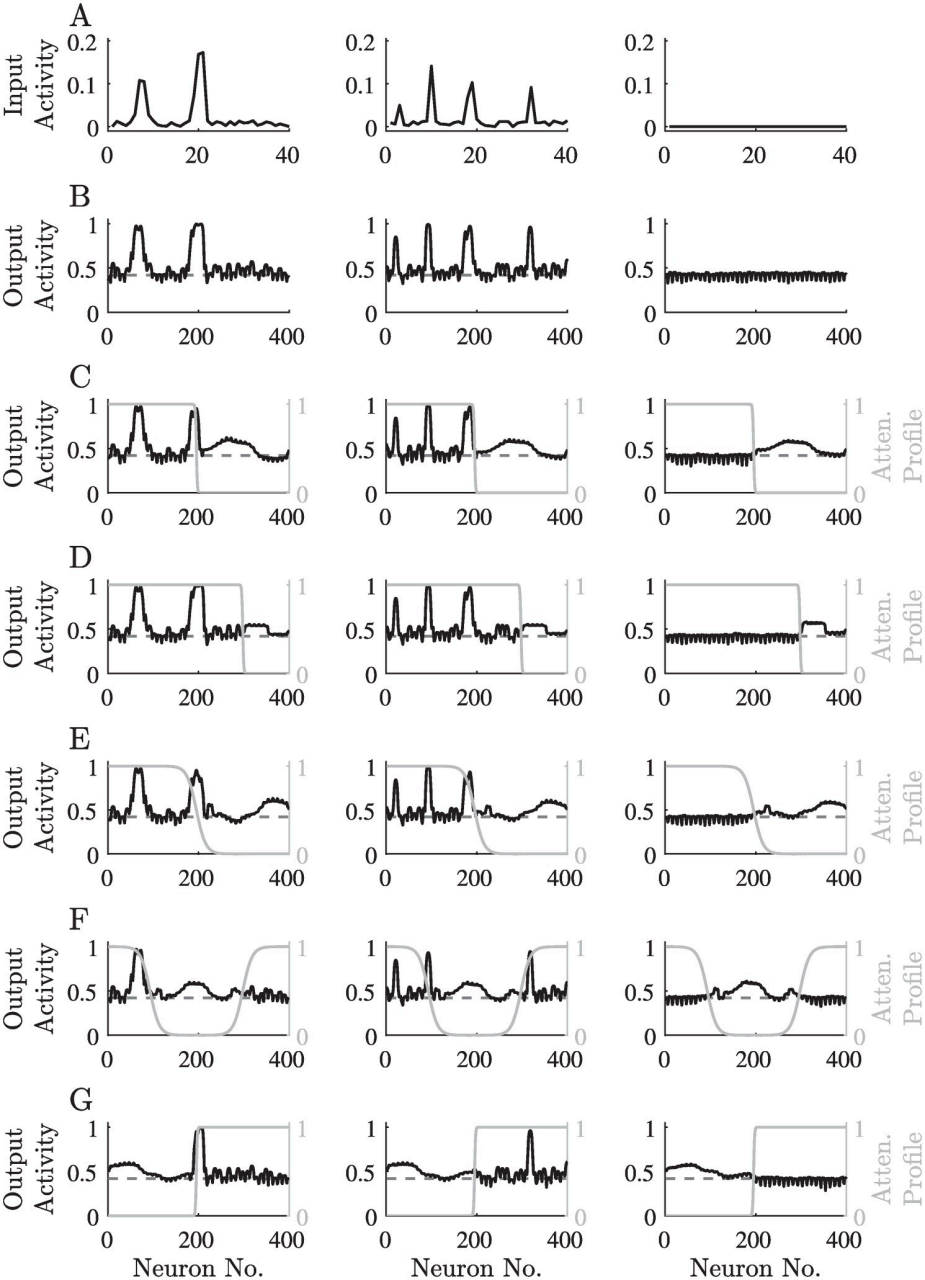
The network’s response to different stimuli before and after sensory deprivation. A: Typical stimuli and a silent stimulus (zero input—right panel). B: The network’s response to the stimuli presented in A. C–G: The network’s response to the stimuli presented in A after training on stimuli with an attenuated frequency range. The attenuation profiles are depicted in gray. The spontaneous activity of the output neurons, defined here as the average activity in response to a silent stimulus before attenuation (as in the right panel of B), is indicated in B–G by a dashed line.

The network’s response to typical stimuli shows tonotopic responses, and the response in the absence of external stimuli is near spontaneous activity (Fig 4A and 4B). We note that the initial feed-forward connectivity was manually tuned to produce a tonotopic mapping (using weak Gaussian profiles with ordered centers). Although the feed-forward connections do change throughout the learning process, the tonotopic organization remains stable. The tonotopic mapping is a well-known property of all auditory processing stages between the cochlea and the auditory cortex in various species, including humans [64–68]. The preservation of the tonotopic organization throughout the learning process is in agreement with biological observations, suggesting that it is created in the embryonic stages of development and is preserved through plasticity processes [69].

**Fig 5:**
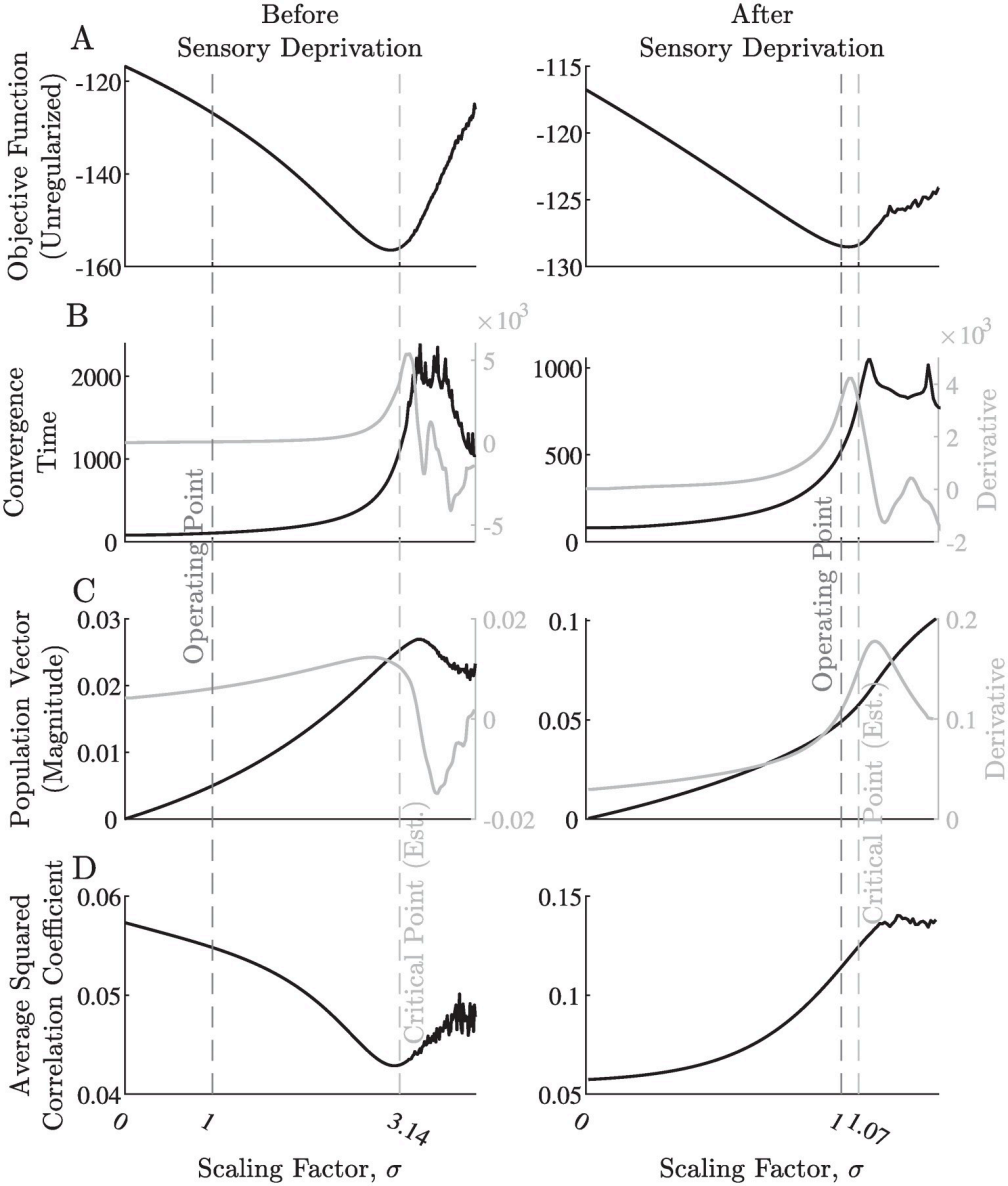
Global measures for different scaling of the recurrent connections. A: The network’s objective function, without the regularization terms. Low values of the objective function correspond to high network’s susceptibility. B: The convergence time of the network dynamics using Euler’s method; i.e., the number of time-steps until the simulation reaches a convergence criterion (see Methods). C: The population vector magnitude. D: The squared correlation coefficient between pairs of output neurons, averaged over all such pairs. All the above measures are displayed for different scaling factors of the recurrent connectivity matrix *K*^*tr*^, as found by the training process; i.e., for each value of the scaling factor *σ*, the different measures were evaluated by replacing the recurrent connectivity matrix with *K* = *σK*^*tr*^. In the left panels, we used the recurrent connectivity matrix *K*^*tr*^ trained on typical stimuli, while in the right panels, we used the recurrent connectivity matrix obtained after sensory deprivation. The operating point is at a scaling factor of 1, namely, the recurrent connectivity the learning process has converged to. The marked critical point (≈3.14 in the left panels and ≈1.07 in the right panels) is the scaling factor for which the spectral radius *ρ*(*K*) of the recurrent connectivity matrix is 4, i.e., 4/*ρ*(*K*^*tr*^). The derivatives of B–C are also displayed for better visualization of transitions in values. The exact values of the objective function and convergence time displayed in A–B are completely arbitrary, therefore these figures should only be considered in a qualitative manner. The attenuation profile’s parameters were *k*_0_ = 20, *β* = 10 (see Fig 2B). For visualization purposes, different panels are displayed on different vertical scales.

We noticed that spatial connectivity profiles hardly change throughout the learning, while their scale changes dramatically. In light of this observation, we quantified several global parameters of the network as a function of the scale of the recurrent connectivity matrix (Fig 5). We also used these measurements to gain insights into the effect of regularization on our results. First, note that the regularization caused the network learning process to converge to down-scaled recurrent interactions compared to the optimal scale in terms of the non-regularized objective function (Fig 5A, dashed vertical lines). This specific scale seems to play a role in determining the proximity of the network dynamics to the critical point. Specifically, the convergence time rises dramatically at this point (Fig 5B), reflecting the well-known phenomenon of “critical slowing down” [70–73]. In addition, at this scale, the population vector’s magnitude rises, reflecting the emergence of non-uniform activity profiles in the absence of a structured input (see Methods and Fig 5C). Finally, the average pairwise correlations obtain a minimum around this scale Fig 5D).

All these results point to the same conclusion—without the regularization, the recurrent connectivity should have been scaled by ≈3.14, such that the spectral radius of the recurrent connectivity matrix would be ≈4. We note that the maximal derivative of the chosen activation function 1/(1 + exp(−*x*)) is ¼. Thus, having the spectral radius of the recurrent connectivity matrix near 4 indicates proximity to the critical point (see Methods). This means that the regularization keeps the recurrent connectivity below its optimal scale (in terms of the entropy term alone), and the network remains subcritical. We note that for different regularization coefficients, the scale of the interactions could obtain different values.

### Sensory deprivation

After the learning was stabilized for normal stimuli, we attenuated the inputs in a certain frequency range (Fig 2B–2F), and let the network’s recurrent connections adapt to the new input statistics. The resulting recurrent connectivity profile among the deprived neurons had a stronger central excitation and a wider inhibition (Fig 3E and 3F and S1 Fig). The stronger recurrent connectivity in the deprived region led to a phase transition, resulting in an inhomogeneous stationary activity pattern independent of the given input (Fig 4C–4G). We interpret those results as “hallucinations”, elicited by the sensory deprivation. Interestingly, the “hallucinations” in our model develop only in the deprived region of the output layer, consistent with certain types of tinnitus [3, 7, 61, 74]. Furthermore, the corresponding activity profile has a single peak, in line with the most common forms of tinnitus [7, 8, 75]. The network’s sensitivity to external inputs in the deprived frequencies is lower, as reflected by the elevated hearing thresholds in the simulated audiograms (S11 Fig).

Following the induction of sensory deprivation, we evaluated the criticality measures once again (Fig 5 right panels, S2 and S3 Figs). The results for the objective function, convergence time and population vector remained qualitatively similar, but the optimal scale moved much closer to 1 (≈1.07). Thus, the network converged to a point much closer to its critical point, compared to its state before the induction of sensory deprivation. Interestingly, the average pairwise correlations now exhibit a maximum rather than a minimum. This finding is qualitatively consistent with the observed increase in synchrony following the induction of tinnitus [76]. We note that following sensory deprivation, the effect of learning on the recurrent connections is not limited to scaling. Hence, the different measures exhibit different patterns in the supercritical domain (above the scale of ≈1.07).

### Regularization effect

As discussed above, to keep the dynamics from crossing into the supercritical domain, we added regularization to the network’s weights. For each type of connectivity matrix (feed-forward and recurrent), we tested regularization both by *ℓ*_1_ and *ℓ*_2_ norms of the connections. Applying *ℓ*_1_ regularization is known to lead to sparse connectivity [77]; however, applying it to the recurrent connectivity matrix ended in nullifying all connections but a few, which were still strong enough to turn the dynamics into the supercritical domain (see S5 and S6 Figs). Because recurrent connectivity is present in most biological neural networks, we chose to focus only on simulations where the recurrent connections were regularized by their *ℓ*_2_ norm. Using either the *ℓ*_1_ or *ℓ*_2_ norm to regularize the feed-forward connectivity did not have a dramatic effect on the results. Since using the *ℓ*_1_ norm leads to a more biological sparse feed-forward connectivity, as found experimentally in the DCN [28], we chose to focus on this option.

The stability of the network’s fixed point is determined by the sign of the eigenvalues of the matrix that controls the linearized dynamics. In this case, the corresponding matrix is (*I* − *GK*), where *K* is the recurrent connectivity matrix and *G* is a diagonal matrix containing the derivatives of the activation function for each output neuron (see Methods). Since the maximal derivative of the chosen activation function (1/(1 + exp(−*x*))) is ¼, the critical point is characterized by having the spectral radius of the recurrent connectivity matrix, *K*, near 4. We used this result as an efficient surrogate to the actual critical point.

**Fig 6:**
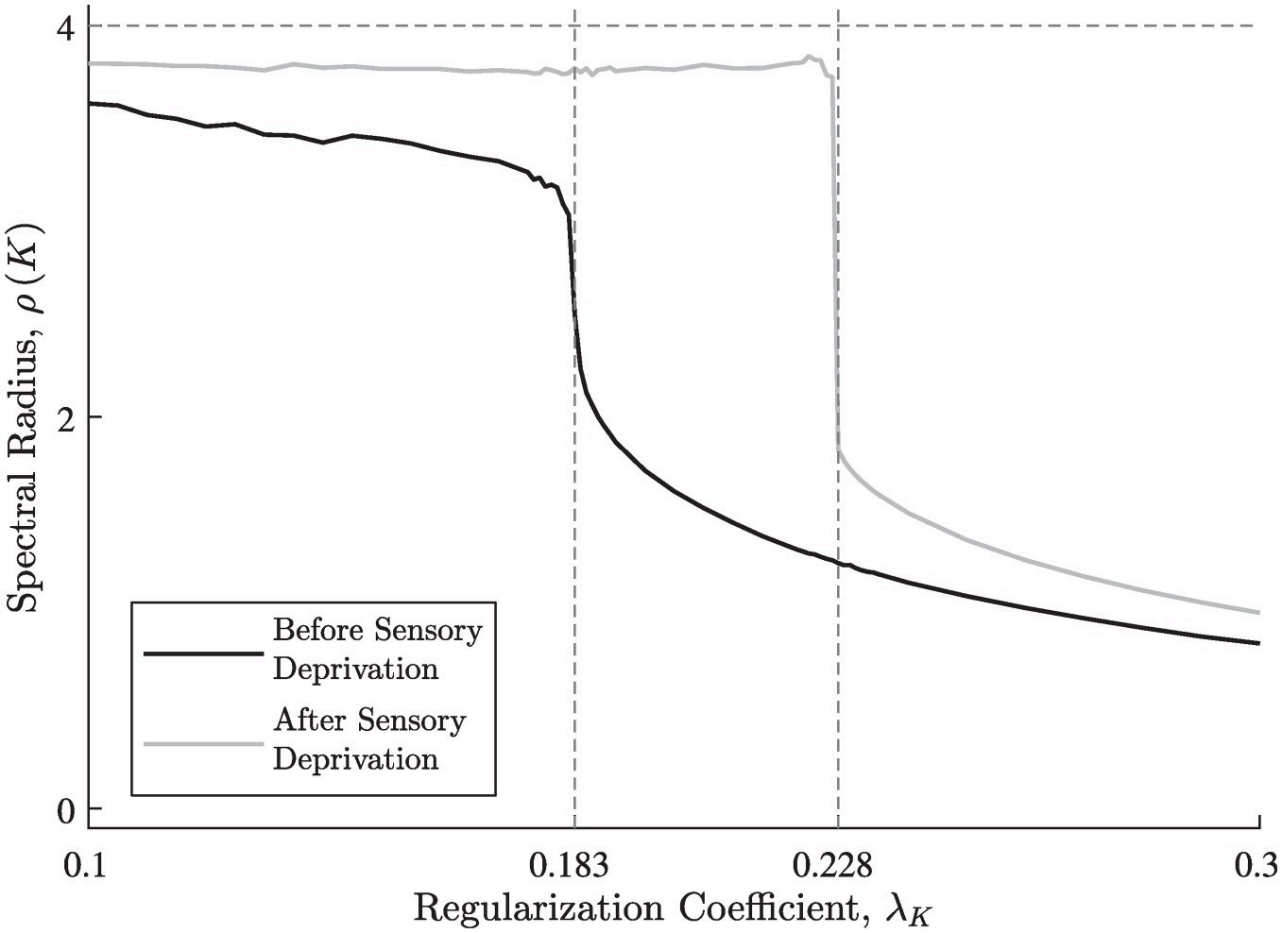
Regularization effect on the spectral radius of the recurrent connectivity matrix. The spectral radius of the recurrent connectivity matrix *K* decreases with the regularization coefficient λ_*K*_, before and after the induction of sensory deprivation. Due to the chosen sigmoidal activation function, the sharp drop in the spectral radius from ≈4 to ≈2 determines the border between near-critical and subcritical dynamics. After the induction of sensory deprivation, this border moves to higher values of the regularization coefficient, hence, creating an interval (from ≈0.183 to ≈0.228) of regularization coefficient values where sensory deprivation causes “hallucinations”. The attenuation profile’s parameters were *k*_0_ = 20, *β* = 10 (see Fig 2B).

In our simulations, the spectral radius of the recurrent connectivity matrix *K* decreased with the respective regularization coefficient λ_*K*_, with a characteristic sharp drop (Fig 6). Generally, the value of λ_*K*_ where this drop occurs depends mainly on the number of output neurons; however, in our simulations, sensory deprivation caused this value to rise. This phenomenon created an interval of λ_*K*_ values, where sensory deprivation drives the dynamics much closer to the critical point, thus, eliciting the hallucination-like responses described before. Interestingly, we found that the results depicted in Fig 6 were robust to changes in the attenuation profile of the inputs (see S4 Fig), suggesting that they depend only on the network’s size and feed-forward connectivity. In all simulations above we used a regularization coefficient near the upper bound of this interval (λ_*K*_ = 0.226), as higher values within the interval tended to yield results more consistent with biological findings, such as the single-peaked “hallucination” profile [8, 75].

## Discussion

In this work, we used an EM approach to train a recurrent neural network to represent simulated auditory stimuli, and examined the effect of input statistics on the evolved representation. For typical inputs, the network developed connectivity patterns and exhibited output responses similar to biological findings regarding the auditory system in general [78–81] and, more specifically, the DCN [27, 28]. Interestingly, sensory deprivation elicited tinnitus-like “hallucinations” in the network, resembling the characteristics of common types of tinnitus [3, 7, 8, 13, 61, 74]. Although we focused here on tinnitus, this qualitative phenomenon is independent of the input modality and can be used to explain how other kinds of “phantom” sensations are caused by neural plasticity and involve the specific region in the sensory input space, which was deprived of input [82, 83].

The DCN is known to receive various non-auditory inputs [30–33]. In particular, somatosensory projections to the DCN are known to be upregulated in tinnitus [22, 34–38], and sensory stimulation modulates the perceived tinnitus in most cases [37, 39, 40]. Conceptually, these findings are in line with the EM approach—strengthening external inputs to a deprived output neuron will tend to increase its entropy. Such upregulation of connections from one sensory modality to another resembles acquired synaesthesia, namely the triggering of sensations in a sensory deprived modality by stimulation of another modality [84]. For example, following visual deafferentation, visual sensations can be elicited by auditory or somatosensory stimuli [85–87]. Indeed, the relationship between tinnitus and acquired somatosensory-auditory synaesthesia was proposed previously [84]. The emergence of such acquired synaesthesia following sensory deprivation has been demonstrated in a network model based on the same EM approach used here [60]. Thus, the proposed computational framework can naturally account for the effect of non-auditory projections.

Nevertheless, the strengthening of feed-forward connections, such as the somatosensory projections, cannot explain the emergence of tinnitus by itself. First, while the perception of tinnitus can be modulated by external feed-forward projections, such projections cannot maintain persistent activity by themselves in the absence of non-auditory stimulation. Second, the perceived tinnitus typically has a distinct spectral profile, whereas a simple enhancement of feed-forward somatosensory inputs would be expected to elicit a homogeneous profile within the deprived frequency range. Recurrent networks, on the other hand, can naturally give rise to and maintain inhomogeneous persistent activity in the absence of external stimulation [88, 89]. Thus, the emergence of tinnitus is likely to rely on changes in recurrent circuitry, although it may also involve additional changes in feed-forward interactions. This study focused on the role of recurrent interactions in the emergence of tinnitus. We note, however, that the corresponding recurrent network may go beyond the DCN and incorporate other brain areas, such as the ventral cochlear nucleus (VCN) and the inferior colliculus (IC), which are known to undergo plastic changes during tinnitus [90–93]. Future work can generalize the current model to also include different non-auditory inputs and model their effect on the perceived tinnitus.

Previous computational models rely on phenomenological homeostasis-driven plasticity to demonstrate tinnitus elicited by sensory deprivation [47–52]. Here, we use an objective-driven plasticity, namely, the main mechanism underlying the network’s plasticity is optimizing an explicit computational goal. Specifically, the network maximizes the entropy of its output, which corresponds to increasing input sensitivity [59]. The general resemblance of our model to biological findings supports the hypothesis that EM serves as a computational objective for primary sensory processing networks in the brain (e.g., [58, 59]). However, as described in the Methods section, the vanilla EM learning rules drive the network into a phase transition. This process may lead the network away from a stable fixed point and into dynamical states with poor information representation. Thus, some regularization should be used to keep the network subcritical. To this end, we used a penalty on the *ℓ*_2_ norm of the recurrent connections as a regularization method, which can be thought of as a kind of homeostatic mechanism [94–98]. Following sensory deprivation, the network increases the gain of its recurrent connectivity to compensate for the attenuated inputs and operates much closer to its critical point, giving rise to tinnitus-like “hallucinations”. In this model, the emergence of tinnitus depends on the interplay between the computational objective and the homeostatic regularization, in contrast to models driven by a single phenomenological homeostatic mechanism. Future studies might employ different types of regularization methods (e.g., firing-rate-based rather than weight-based) and examine their effect on the dynamics of the network.

While most of the hyper-parameters of the model can be chosen arbitrarily without having any qualitative effect on the results, the regularization coefficient for the recurrent connectivity, λ_*K*_, is an exception; if it is too small, numerical instabilities might accidentally drive the network into a supercritical domain, but if it is too large, the network will always remain subcritical. In the first case, the output may no longer be dependent on the input, while in the second case, the input may have little effect on the output—in both cases, moving away from the critical point leads to poor sensitivity. In practice, there is a specific range of values which yields the qualitative results demonstrated in this paper (see Fig 6) and, according to our observations, it is independent on the chosen attenuation profile (see S4 Fig). Here, we used a grid search to find the corresponding range, and the results were obtained using a near-maximal value within it. This choice maximized the cost of regularization relative to the EM objective, while still allowing a sensory deprivation to drive the dynamics away from the subcritical regime. This choice of λ_*K*_ has driven the network towards single-peaked “hallucinations”, matching empirical findings [8, 75].

These results are interesting to discuss in light of a plethora of studies from recent years, suggesting near-critical dynamics in biological neural networks across various scales, from neuronal cultures to large-scale human brain activity [99–107]. In particular, it is proposed that healthy neural dynamics are poised near a critical point, yet within the subcritical domain [108]. Changes in the input statistics can drive the network to transition into supercritical dynamics, which may manifest as hallucinations. Our study portrays a concrete, albeit simplified, network model that experiences a transition from healthy to pathological neural dynamics as a consequence of inherent plasticity and sensory deprivation. We note that the network dynamics here are too simplified to enable a direct comparison with the rich dynamics observed in cortical networks and with common hallmarks of criticality (e.g., [99]).

An illuminating perspective on the emergence of hallucinations, such as tinnitus, as a consequence of sensory deprivation comes from the framework of Bayesian inference [109–111]. According to this framework, sensory systems generate perception by combining the incoming stimuli with prior expectations in a way that takes into account the relative uncertainty of each. Under sensory deprivation, the uncertainty about the input is very large; hence, the weight of the prior expectations becomes more dominant. This process may eventually lead to a state in which prior expectations dominate perception, which can be interpreted as a hallucination [112]. If this perception is maintained long enough, it will turn into a strong prior by itself, thus, giving rise to a chronic hallucination—namely, tinnitus [110]. Although our model does not use the Bayesian framework explicitly, it can be thought of in similar terms. Here, the prior expectations are effectively encoded in the evolved recurrent connectivity. Under sensory deprivation, these recurrent interactions dominate the network’s response and can be thought of as an enhanced prior. The advantage of the model described here lies in its mechanistic nature, namely, that it is cast in the language of neuronal networks with long-term plasticity of recurrent interactions. Thus, it can be more straightforward to interpret and compare to experimental data.

It is important to note that this model is relatively simplified in terms of the network architecture and dynamics. For example, the steady-state response used here reflects an assumption of slowly modulated inputs (compared to the network dynamics), which is usually reasonable in the case of the auditory system, but it does not hold for all cases. As a consequence, the model cannot fully capture some of the underlying details, such as the spectral response properties of DCN neurons and dynamical aspects like bursting and synchrony; however, such simplifications are currently necessary to allow the derivation of EM-based learning rules for the recurrent connections [57]. Developing suitable EM-based learning rules for non-stationary inputs and outputs is an interesting and challenging task by itself, and its application to scenarios of sensory deprivation may lead to further insights, but such derivation lies beyond the scope of the current work. We believe that the underlying principle of EM leading to hallucinations under sensory deprivation does not depend on such details. Future work can use the same computational principles with a more biologically-detailed network model to better account for other aspects as well.

To summarize, we have demonstrated how the EM approach can be used as a model of early auditory processing and the phenomenon of tinnitus. Previous works have demonstrated that EM-based neural networks can serve as models for early visual processing [58, 59] and the phenomenon of synaesthesia [60]. We believe that this framework can be used for modeling other modalities and phenomena as well. It is also important to extend this framework to more biologically plausible network models, which could account for more detailed aspects of the underlying neural dynamics.

## Methods

### The model

We modeled an early auditory processing neural network (e.g., the DCN) using the overcomplete recurrent EM neural network described in [57], with the addition of regularization on strong connectivity.

#### Network architecture and dynamics

Our system is composed of *M* input neurons, **x**, and *N* output neurons, **s**. Each output neuron’s activity through time is given by the dynamic equation:

$$\begin{array}{c} \tau \frac{\text{d}s_{i}}{\text{d}t}=-s_{i}+g(\sum_{j=1}^{M}W_{ij}x_{j}+\sum_{k=1}^{N}K_{ik}s_{k}-T_{i}), \end{array}$$ (1)

where *W* is the feed-forward connectivity matrix, *K* is the recurrent connectivity matrix, *T* are the output neurons’ thresholds, and *g*(*x*) = 1/(1 + exp(−*x*)) is the activation function of the neurons. For overcomplete transformations, we assume *M* < *N* (Fig 1).

The fixed points of Eq 1 are given implicitly by:

$$\begin{array}{c} s_{i}=g(\sum_{j=1}^{M}W_{ij}x_{j}+\sum_{k=1}^{N}K_{ik}s_{k}-T_{i}). \end{array}$$ (2)

These fixed points are stable iff all of the eigenvalues of the linearized dynamics matrix (*I* − *GK*) have positive real parts [59] (*G* is a diagonal matrix defined by $G_{ij}\equiv \delta_{ij}g^{\prime }(\sum_{j=1}^{M}W_{ij}x_{j}+\sum_{k=1}^{N}K_{ik}s_{k}-T_{i})$). Since the values of *G* are upper-bounded by max_*x*_ *g*′ (*x*) = ¼, for a matrix *K* with eigenvalues <4, the fixed points are indeed stable. In practice, when fixed points exist at all, there will usually be only one such stable fixed point.

Numerically, the steady state can be found via integrating Eq 1 using Euler’s method for a long time-period until the activities stabilize; however, this method is highly inefficient. In this work, we found the steady state by solving Eq 2 directly using the Newton-Raphson method.

When the eigenvalues of *K* are near 4, the eigenvalues of (*I* − *GK*) might get close to zero. Crossing this point will result in instability of the fixed point and a phase transition. Near this phase transition, the decrease in the eigenvalues of (*I* − *GK*) will cause the effective time constants to rise—a phenomenon termed “critical slowing down”. To gain some insight into the actual effective time constant, we evaluated the convergence time of Eq 1 by integrating it using Euler’s method, and counting the number of time-steps until a convergence criterion was met.

Furthermore, such a phase transition is expected to be characterized by a spontaneous symmetry breaking [113], which can be measured by several metrics. Here, we used the population vector for that purpose, calculated as $\frac{1}{N}\sum_{k=1}^{N}s_{k}e^{i\phi_{k}}$ where *ϕ*_*k*_ ≡ 2*πk*/*N* and *k* is the index of the output neuron. Although in our case the boundary conditions are not periodic, we assume their effect to be negligible since *N* ≫ 1 and treat the preferred frequencies of the neurons as preferred angles.

#### Learning rules

The goal of the network is to find the set {W*, K*, T*} which maximizes the entropy *H*(**s**) of the steady state outputs. To do so, we used the objective function described in [57], with additional regularization terms on the *ℓ*_1_ and *ℓ*_2_ norms of *W* and *K*, respectively:

$$\begin{array}{c} \varepsilon \equiv -\frac{1}{2}{\left\langle \text{log}\;(\chi^{T}\chi )\right\rangle }_{x}+\lambda_{W}\sum_{i,j}|W_{ij}|+\frac{\lambda_{K}}{2}\sum_{i,k}K_{ik}^{2}, \end{array}$$ (3)

where $\chi_{ij}\equiv \frac{\partial s_{i}}{\partial x_{j}}$ is the Jacobian of the transformation given by *χ* = *ϕW*, and *ϕ* ≡ (*I* − *GK*)^−1^ *G* [57].

This objective function, without the regularization terms, would lead to an increase in the singular values of *χ*. One way to achieve that goal is to decrease the eigenvalues of (*I* − *GK*) to zero, which may lead one of them to turn slightly negative due to numerical errors. This will result in instability of the fixed point and a phase transition, as discussed above. The goal of the regularization terms is to prevent this phenomenon, which is a general property of unregularized entropy maximization systems of continuous variables [114].

The learning rules were derived using the gradient descent method, as in [57]:

$$\begin{array}{ll} \Delta W & \equiv -\eta \frac{\partial \varepsilon }{\partial W}=\eta \left({\langle \phi^{T}\left({\left(\chi^{+}\right)}^{T}+\text{y}{\text{x}}^{T}\right)\rangle }_{\text{x}}-\lambda_{W}S\left(W\right)\right)\\ \Delta K & \equiv -\eta \frac{\partial \varepsilon }{\partial K}=\eta \left({\langle \phi^{T}\left(\chi \chi^{+}+\text{y}{\text{s}}^{T}\right)\rangle }_{\text{x}}-\lambda_{K}K\right)\\ \Delta T & \equiv -\eta \frac{\partial \varepsilon }{\partial T}=\eta {\langle -\phi^{T}\text{y}\rangle }_{\text{x}}, \end{array}$$ (4)

where $y_{l}\equiv {\left(\chi \chi^{+}\phi \right)}_{ll}\frac{g^{\prime \prime }(h_{l})}{{\left(g^{\prime }(h_{l})\right)}^{3}}$, $h_{l}\equiv \sum_{j=1}^{M}W_{lj}x_{j}+\sum_{k=1}^{N}K_{lk}s_{k}-T_{l}$, *S*(*A*) is defined by (*S*(*A*))_*ij*_ ≡ sign (*A*_*ij*_) and *χ*^+^ stands for the pseudo-inverse of *χ* (in the overcomplete case used here, *χ*^+^ = (*χ*^*T*^ *χ*) *χ*^*T*^).

### Auditory inputs

The input stimuli were chosen according to certain heuristics to emulate the system’s response to tones of varying frequencies and amplitudes. Each input sample embodies the reaction of the auditory hair cells to a combination of tones of certain frequencies. As the cochlea maps the frequencies on a logarithmic scale, we assumed each pair of adjacent input neurons, representing inner hair cells, to represent equally log-spaced frequencies. The input profile for a pure tone is centered on the neuron that best matches that frequency, and drops off to neighboring neurons to form a narrow Gaussian response curve. The frequency of each pure tone was chosen at random with a uniform distribution (in the log-spaced frequency domain) within the permitted range. The amplitude of each pure tone was randomly drawn from a uniform distribution, reflecting the unimodal distribution of the logarithms of amplitudes in natural sounds (e.g., [115]). Other unimodal distributions, e.g., the normal distribution, may also be used to model the logarithms of the amplitudes. To account for the logarithmic response of hair cells and the auditory nerve to different amplitudes [116, 117], we modeled the distribution of the logarithms of the amplitudes rather than that of the raw amplitudes. In addition to the input response, all neurons feature some spontaneous random activity that is irrespective of the inputs, to model the neurons’ reaction to background noises and non-stimulated motion of the hair cells (Fig 2A).

The amplitudes of natural sounds are not uniformly distributed, loud sounds being exponentially less common; however, the response of the inner hair cells is determined not only by the absolute amplitude of the sound, but also by the reactivity of the basilar membrane, as controlled by the outer hair cells. This serves as an automatic gain control mechanism, giving the inner hair cells use of their full motion capacity for normal inputs. Therefore, we hold the uniform distribution to be a good approximation to the output of the inner hair cells when presented with natural sounds [118, 119].

To model sensory deprivation, we attenuated a part of the frequency domain by applying a (monotonically decreasing) sigmoid envelope to all stimuli. The choice of attenuating the higher frequencies in most attenuation profiles was based on the most common type of hearing loss [120, 121], but attenuation was also applied to other frequency bands (Fig 2B–2F).

### Implementation details

#### Input generation

Each input sample was composed of up to 5 different tones, uniformly distributed in the input domain. The response to each tone was a Gaussian, with a folded-normally distributed standard deviation (the standard deviations themselves have a standard deviation of half the input domain) and a uniformly distributed amplitude between 7 and 10 (arbitrary units). An additive uniformly distributed noise between 0 and 1 was added to each simulated input sample. Finally, all input samples were divided by twice the highest activation obtained over all samples and input neurons, such that the new activations were in the range [0, 0.5].

#### Attenuation profiles

Input attenuation of high frequencies was simulated by multiplying each input neuron’s activity by a factor between 0 and 1. This factor was chosen according to a sigmoid function: *a*(*k*) = 1/(1 + exp(−*β*(*k*_0_ − *k*))), where *k* is the input neuron’s index, *k*_0_ represents the cutoff frequency in the input neurons domain (analogous to the log-scaled frequency domain) and *β* controls the attenuation profile’s steepness. Here we chose *k*_0_ to be at either ½ (Fig 2B, 2D and 2F) or ¾ (Fig 2C) of the number of input neurons, and *β* to be either 10 (Fig 2B and 2C), 1 (Fig 2D) or -10 (a non-inverted sigmoid; Fig 2F). To simulate a hearing loss at a certain frequency band, we combined two sigmoidal functions to get the attenuation profile: *a*(*k*) = 1 − (1 − 1/(1 + exp(−*β*(*k*_1_ − *k*)))) ⋅ (1 − 1/(1 + exp(−*β*(*k* − *k*_2_)))), where *k*_1_ and *k*_2_ are the edges of the frequency band, defined similarly to *k*_0_ in the previous cases. Here, we chose *k*_1_ and *k*_2_ to be at ¼ and ¾ of the number of input neurons, respectively, and *β* to be 1 (Fig 2E).

#### Training schedule and hyper-parameters

The network was trained in an on-line manner using 1,000,000 samples randomly drawn as described in the Input generation subsection. The training process was divided into three phases:

1. **Feed-forward training**: Only the feed-forward connections (*W*) and the thresholds (*T*) were trained using unattenuated inputs for 50,000 iterations. The learning rate was *η* = 0.1 and the feed-forward regularization coefficient was set to λ_*W*_ = 0.001. During this phase the recurrent connections were set to zero.
2. **Recurrent training**: Only the recurrent connections (*K*) were trained using unattenuated inputs for 1,000,000 iterations. The learning rate was *η* = 0.001 and the regularization coefficient was λ_*K*_ = 0.226 (see Regularization effect). During training, auto-synapses (from an output neuron to itself) were manually truncated to zero.
3. **Attenuated inputs training**: The training continued exactly as in the previous recurrent training phase (phase 2) for another 1,000,000 iterations, but now the inputs were attenuated.

We note that the different number of iterations in each phase was chosen to be large enough to implicate full convergence of the learning process. In practice, the learning usually converges after much fewer iterations.

While the second learning phase was meant to simulate a normal development of the recurrent connectivity prior to the sensory deprivation, similar results to those displayed throughout the paper are also obtained without it (see S7–S10 Figs).

## Supporting information

S1 Fig The network’s recurrent connectivity before and after sensory deprivation for different attenuation profiles. Each row of panels depicts the recurrent connectivity matrix and its average row profile after sensory deprivation, averaged separately for neurons in the deprived zone and the non-deprived zone. Each row match the attenuation profiles from panels C–F in Fig 2, respectively. See Fig 3 for further details.
(TIF)

S2 Fig Global measures for different scaling of the recurrent connections. A: The network’s objective function, without the regularization terms. B: The convergence time of the network dynamics using Euler’s method. C: The population vector magnitude. D: The squared correlation coefficient between pairs of output neurons, averaged over all such pairs. All the above measures are displayed for different scaling factors of the recurrent connectivity matrix *K*^*tr*^, as found by the training process; i.e., for each value of the scaling factor *σ*, the different measures were evaluated by replacing the recurrent connectivity matrix with *K* = *σK*^*tr*^. The recurrent connectivity matrices used here were obtained after sensory deprivation. The left and right panels correspond to attenuation profiles with *k*_0_ = 30, *β* = 10 and *k*_0_ = 20, *β* = 1, respectively (Fig 2C and 2D). The operating point is at a scaling factor of 1, namely, the recurrent connectivity the learning process has converged to. The marked critical point is the scaling factor for which the spectral radius *ρ*(*K*) of the recurrent connectivity matrix is 4, i.e., 4/*ρ*(*K*^*tr*^). See Fig 5 for further details.
(TIF)

S3 Fig Global measures for different scaling of the recurrent connections. A: The network’s objective function, without the regularization terms. B: The convergence time of the network dynamics using Euler’s method. C: The population vector magnitude. D: The squared correlation coefficient between pairs of output neurons, averaged over all such pairs. All the above measures are displayed for different scaling factors of the recurrent connectivity matrix *K*^*tr*^, as found by the training process; i.e., for each value of the scaling factor *σ*, the different measures were evaluated by replacing the recurrent connectivity matrix with *K* = *σK*^*tr*^. The recurrent connectivity matrices used here were obtained after sensory deprivation. The left and right panels correspond to the last two attenuation profiles from Fig 2 (panels E and F, respectively). The operating point is at a scaling factor of 1, namely, the recurrent connectivity the learning process has converged to. The marked critical point is the scaling factor for which the spectral radius *ρ*(*K*) of the recurrent connectivity matrix is 4, i.e., 4/*ρ*(*K*^*tr*^). See Fig 5 for further details.
(TIF)

S4 Fig Regularization effect on the spectral radius of the recurrent connectivity matrix. The spectral radius, *ρ*(*K*), of the recurrent connectivity matrix *K* as a function of the regularization coefficient λ_*K*_, before and after the induction of different sensory deprivation profiles. See Fig 6 for further details.
(TIF)

S5 Fig The network’s recurrent connectivity before and after sensory deprivation using *ℓ*_1_ regularization. A–C: The recurrent connectivity matrix and its average row profile and connectivity distribution, before sensory deprivation. D–F: Same as A–C, but after sensory deprivation. In E, the row profiles were averaged separately for neurons in the deprived zone and the non-deprived zone. The attenuation profile’s parameters were *k*_0_ = 20, *β* = 10 (see Fig 2B). See Fig 3 for further details.
(TIF)

S6 Fig The network’s response to different stimuli before and after sensory deprivation using *ℓ*_1_ regularization. A: Typical stimuli and a silent stimulus (zero input—right panel). B: The network’s response to the stimuli presented in A. C: The network’s response to the stimuli presented in A after training on stimuli with attenuated high frequencies. The attenuation profile is depicted in gray. The spontaneous activity of the output neurons, defined here as the average activity in response to a silent stimulus before attenuation (as in the right panel of B), is indicated in B–C by a dashed line. See Fig 4 for further details.
(TIF)

S7 Fig The network’s recurrent connectivity after sensory deprivation without pretraining the recurrent connections on normal stimuli. A: The recurrent connectivity matrix. B: The average row profile of the recurrent connectivity matrix, averaged separately for neurons in the deprived zone and the non-deprived zone. The attenuation profile’s parameters were *k*_0_ = 20, *β* = 10 (see Fig 2B). See Fig 3 for further details.
(TIF)

S8 Fig The network’s response to different stimuli before and after sensory deprivation, without pretraining the recurrent connections on normal stimuli. A: Typical stimuli and a silent stimulus (zero input—right panel). B: The network’s response to the stimuli presented in A after training only the feed-forward connections. C: The network’s response to the stimuli presented in A after training on stimuli with attenuated high frequencies. The attenuation profile is depicted in gray. The spontaneous activity of the output neurons, defined here as the average activity in response to a silent stimulus before attenuation (as in the right panel of B), is indicated in B–C by a dashed line. See Fig 4 for further details.
(TIF)

S9 Fig Global measures for different scaling of the recurrent connections, without pretraining the recurrent connections on normal stimuli. A: The network’s objective function, without the regularization terms. B: The convergence time of the network dynamics using Euler’s method. C: The population vector magnitude. D: The squared correlation coefficient between pairs of output neurons, averaged over all such pairs. All the above measures are displayed for different scaling factors of the recurrent connectivity matrix *K*^*tr*^, as found by the training process; i.e., for each value of the scaling factor *σ*, the different measures were evaluated by replacing the recurrent connectivity matrix with *K* = *σK*^*tr*^. The recurrent connectivity matrix used here was obtained after sensory deprivation. The attenuation profile used had the parameters *k*_0_ = 20, *β* = 10. The operating point is at a scaling factor of 1, namely, the recurrent connectivity the learning process has converged to. The marked critical point is the scaling factor for which the spectral radius *ρ*(*K*) of the recurrent connectivity matrix is 4, i.e., 4/*ρ*(*K*^*tr*^). See Fig 5 for further details.
(TIF)

S10 Fig Regularization effect on the spectral radius of the recurrent connectivity matrix, without pretraining the recurrent connections on normal stimuli. The spectral radius, *ρ*(*K*), of the recurrent connectivity matrix *K* as a function of the regularization coefficient λ_*K*_, after the induction of sensory deprivation. See Fig 6 for further details.
(TIF)

S11 Fig Simulated audiograms for different attenuation profiles. A: A simulated audiogram without sensory deprivation. B–F: Simulated audiograms for different attenuation profiles, matching the ones in Fig 2B–2F, respectively. To simulate subjective hearing thresholds, the threshold of each frequency represents the input activity required to produce a difference of 0.01 (measured by *ℓ*_∞_-norm) between a silent input and an input where only the specific frequency is active. The thresholds were found using the bisection method in the interval [0, 100], with a tolerance of 10^−6^.
(TIF)
