# Supplementary figures and images for: Tinnitus-like “hallucinations” elicited by sensory deprivation in an entropy maximization recurrent neural network
Source: PLoS Comput Biol. 2021 Dec 8;17(12):e1008664. doi: 10.1371/journal.pcbi.1008664 (PMC8687580; doi:10.1371/journal.pcbi.1008664)

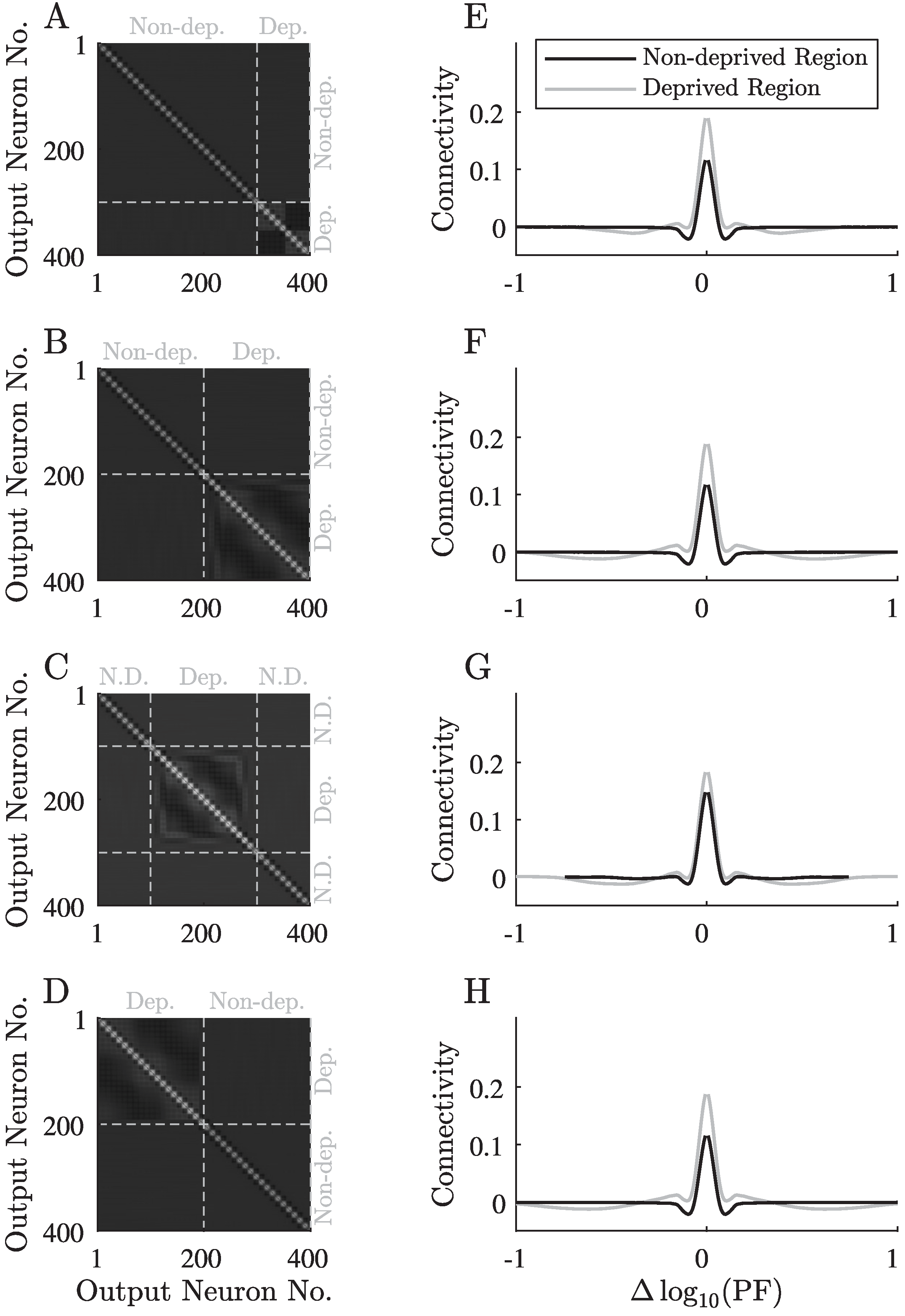

Supplement: S1 Fig — Each row of panels depicts the recurrent connectivity matrix and its average row profile after sensory deprivation, averaged separately for neurons in the deprived zone and the non-deprived zone. Each row match the attenuation profiles from panels C–F in Fig 2, respectively. See Fig 3 for further details. (TIF) [file pcbi.1008664.s001.tif]

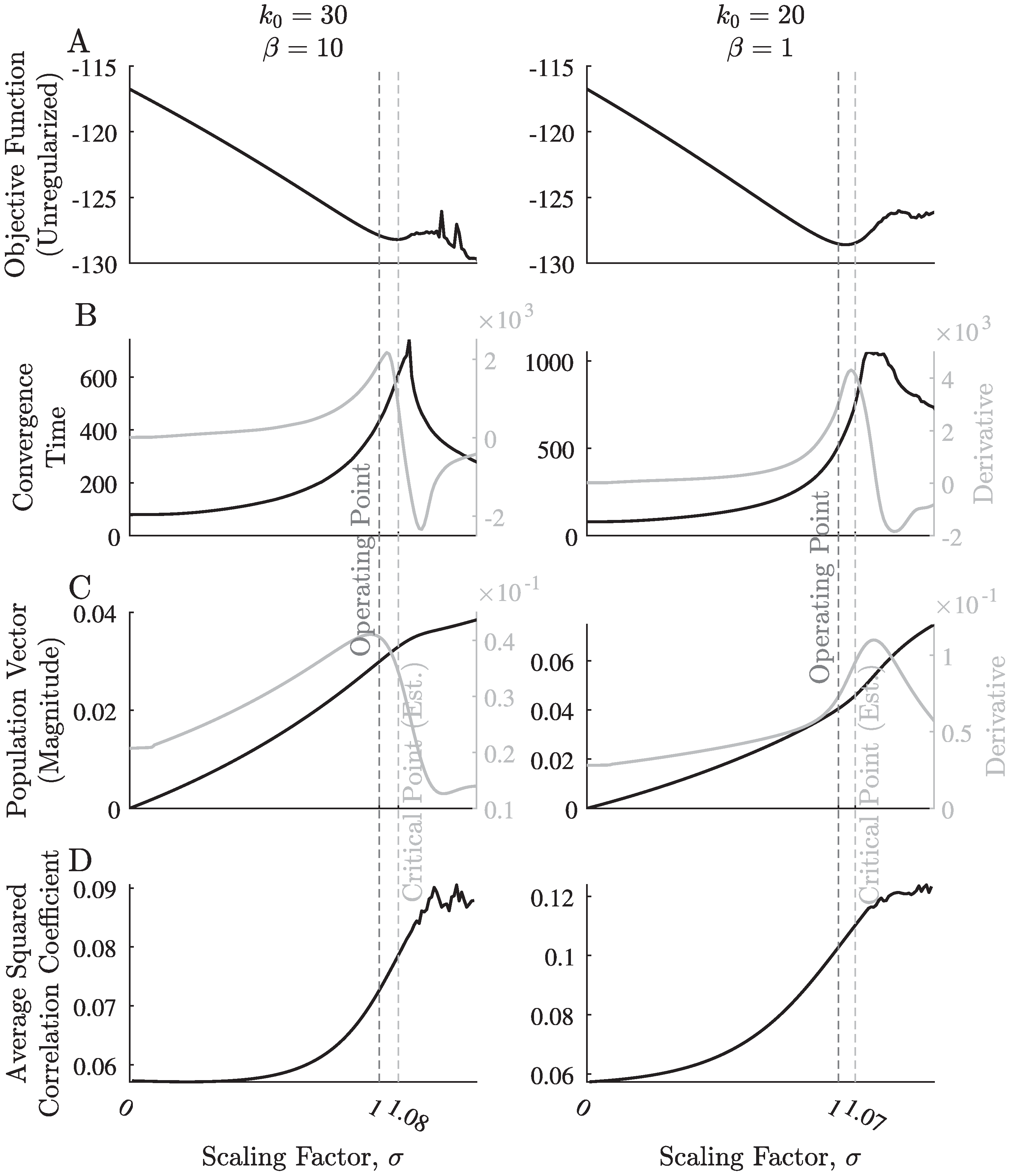

Supplement: S2 Fig — A: The network’s objective function, without the regularization terms. B: The convergence time of the network dynamics using Euler’s method. C: The population vector magnitude. D: The squared correlation coefficient between pairs of output neurons, averaged over all such pairs. All the above measures are displayed for different scaling factors of the recurrent connectivity matrix Ktr, as found by the training process; i.e., for each value of the scaling factor σ, the different measures were evaluated by replacing the recurrent connectivity matrix with K = σKtr. The recurrent connectivity matrices used here were obtained after sensory deprivation. The left and right panels correspond to attenuation profiles with k0 = 30, β = 10 and k0 = 20, β = 1, respectively (Fig 2C and 2D). The operating point is at a scaling factor of 1, namely, the recurrent connectivity the learning process has converged to. The marked critical point is the scaling factor for which the spectral radius ρ(K) of the recurrent connectivity matrix is 4, i.e., 4/ρ(Ktr). See Fig 5 for further details. (TIF) [file pcbi.1008664.s002.tif]

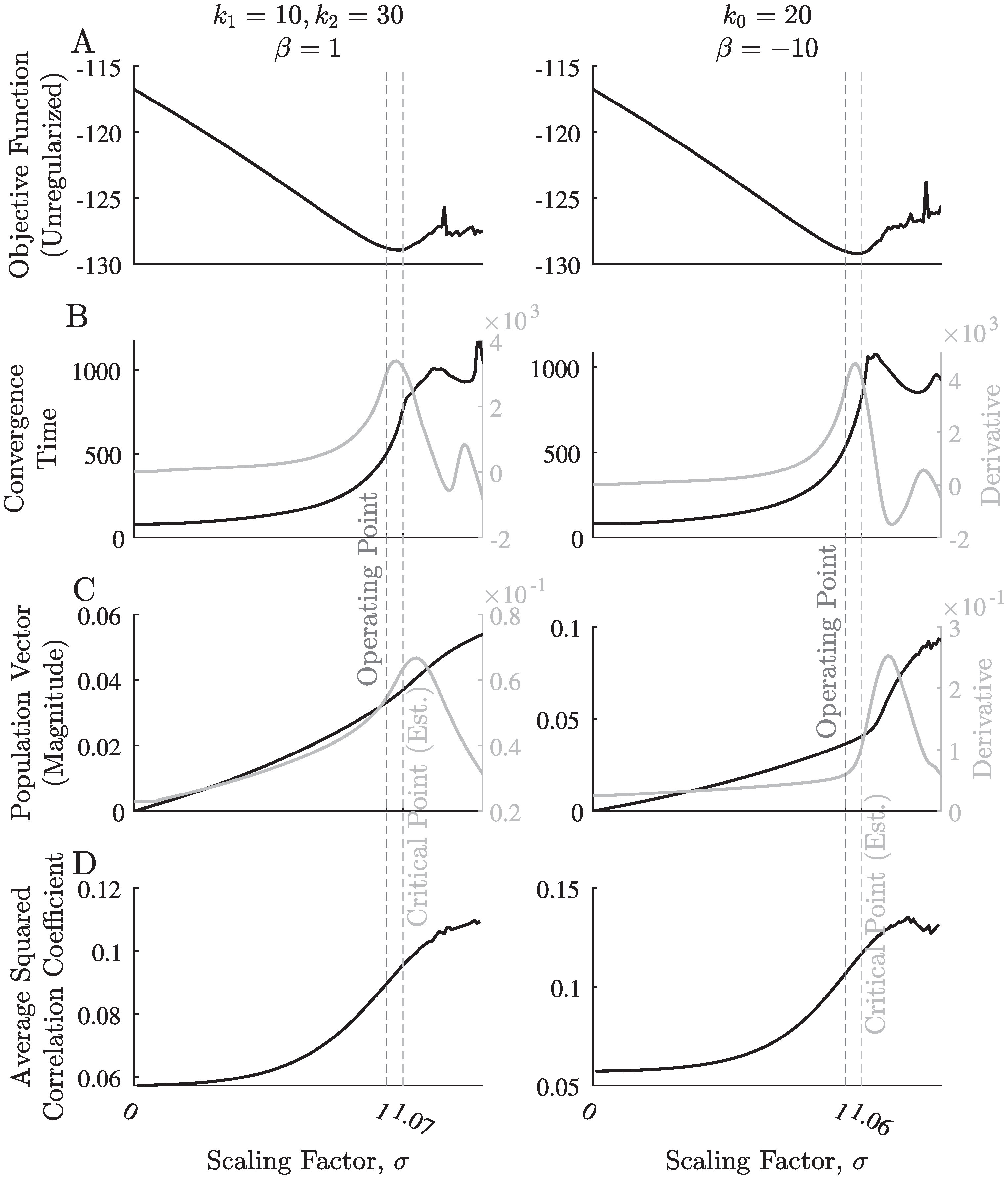

Supplement: S3 Fig — A: The network’s objective function, without the regularization terms. B: The convergence time of the network dynamics using Euler’s method. C: The population vector magnitude. D: The squared correlation coefficient between pairs of output neurons, averaged over all such pairs. All the above measures are displayed for different scaling factors of the recurrent connectivity matrix Ktr, as found by the training process; i.e., for each value of the scaling factor σ, the different measures were evaluated by replacing the recurrent connectivity matrix with K = σKtr. The recurrent connectivity matrices used here were obtained after sensory deprivation. The left and right panels correspond to the last two attenuation profiles from Fig 2 (panels E and F, respectively). The operating point is at a scaling factor of 1, namely, the recurrent connectivity the learning process has converged to. The marked critical point is the scaling factor for which the spectral radius ρ(K) of the recurrent connectivity matrix is 4, i.e., 4/ρ(Ktr). See Fig 5 for further details. (TIF) [file pcbi.1008664.s003.tif]

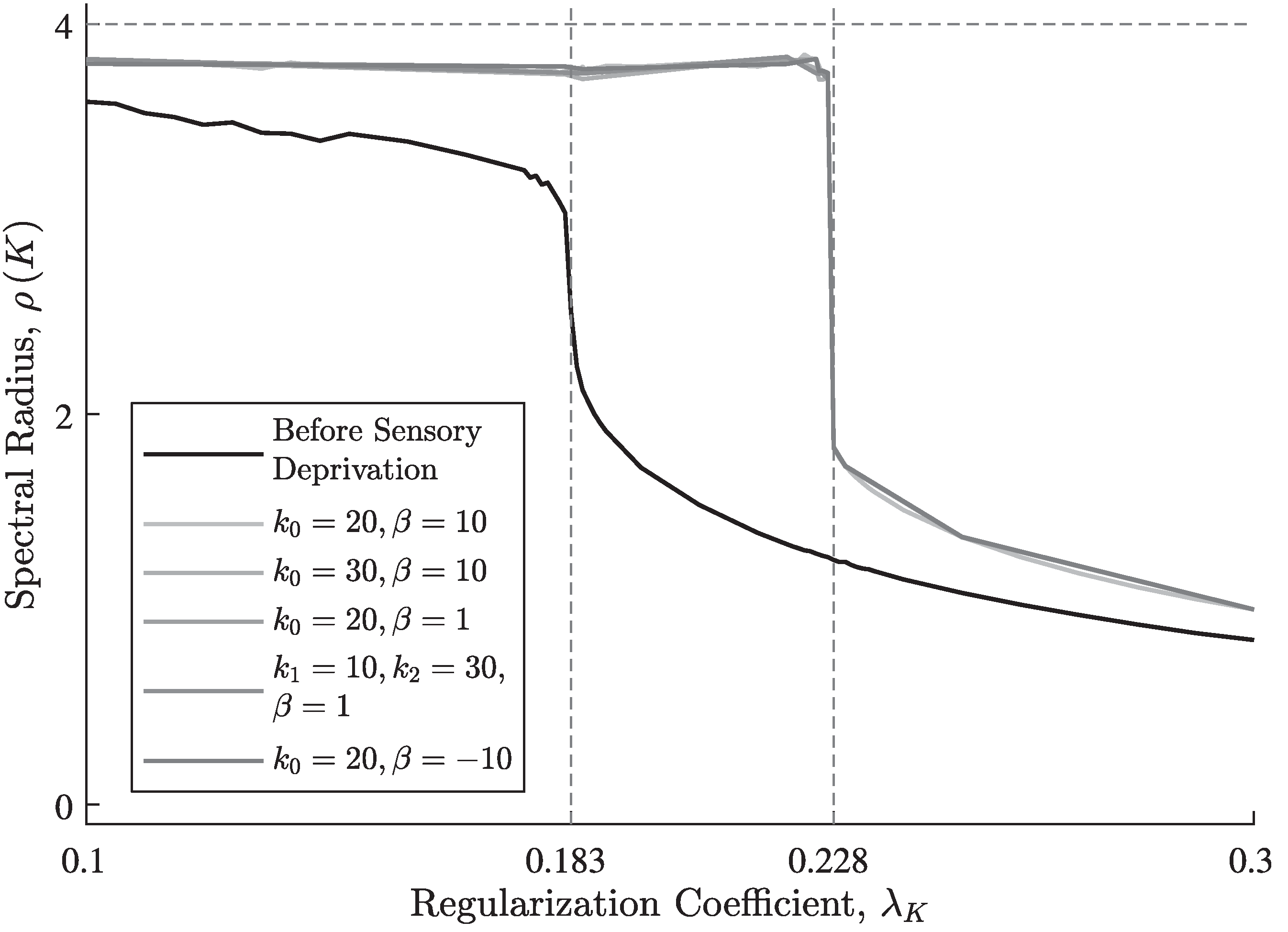

Supplement: S4 Fig — The spectral radius, ρ(K), of the recurrent connectivity matrix K as a function of the regularization coefficient λK, before and after the induction of different sensory deprivation profiles. See Fig 6 for further details. (TIF) [file pcbi.1008664.s004.tif]

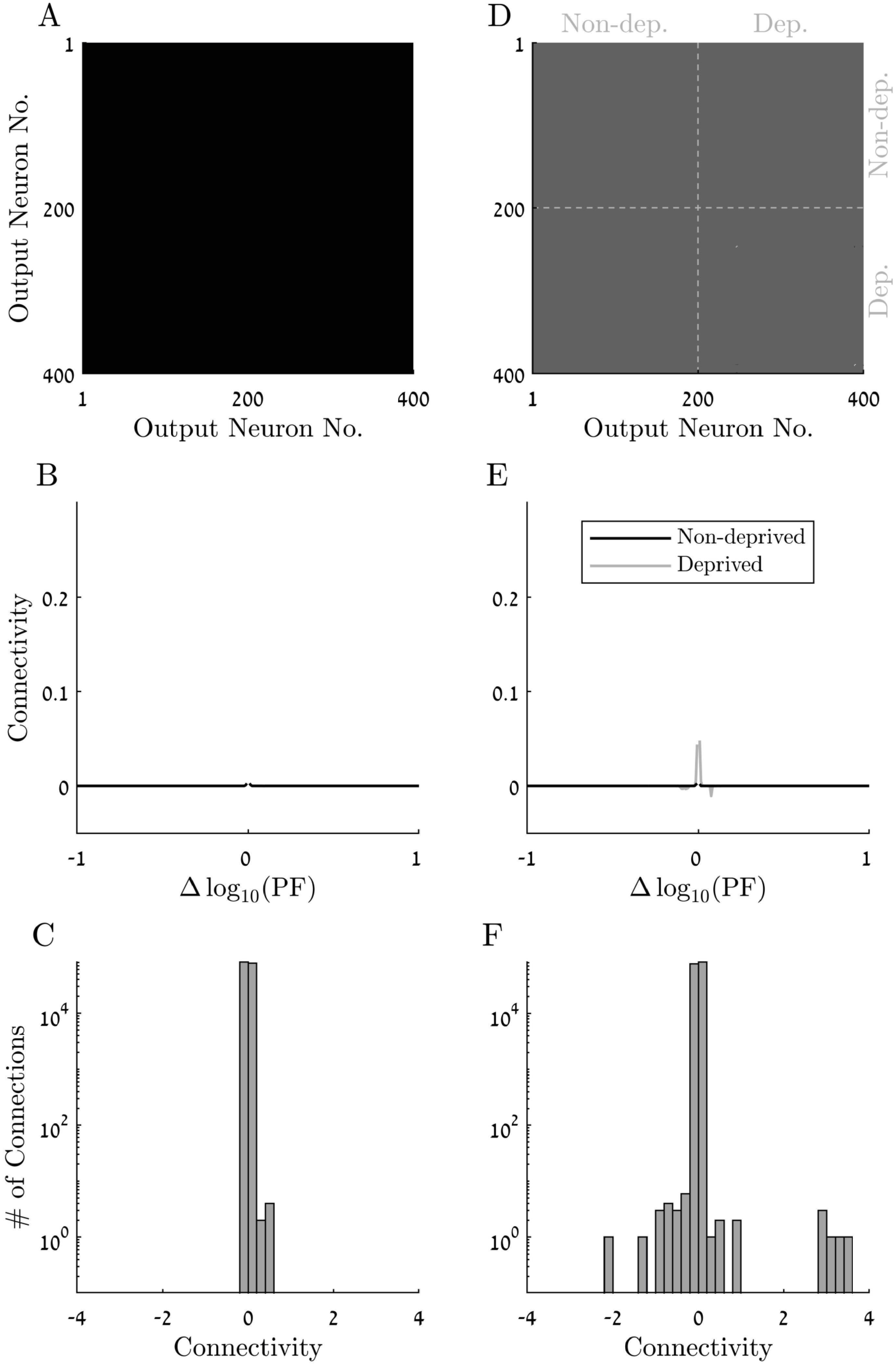

Supplement: S5 Fig — A–C: The recurrent connectivity matrix and its average row profile and connectivity distribution, before sensory deprivation. D–F: Same as A–C, but after sensory deprivation. In E, the row profiles were averaged separately for neurons in the deprived zone and the non-deprived zone. The attenuation profile’s parameters were k0 = 20, β = 10 (see Fig 2B). See Fig 3 for further details. (TIF) [file pcbi.1008664.s005.tif]

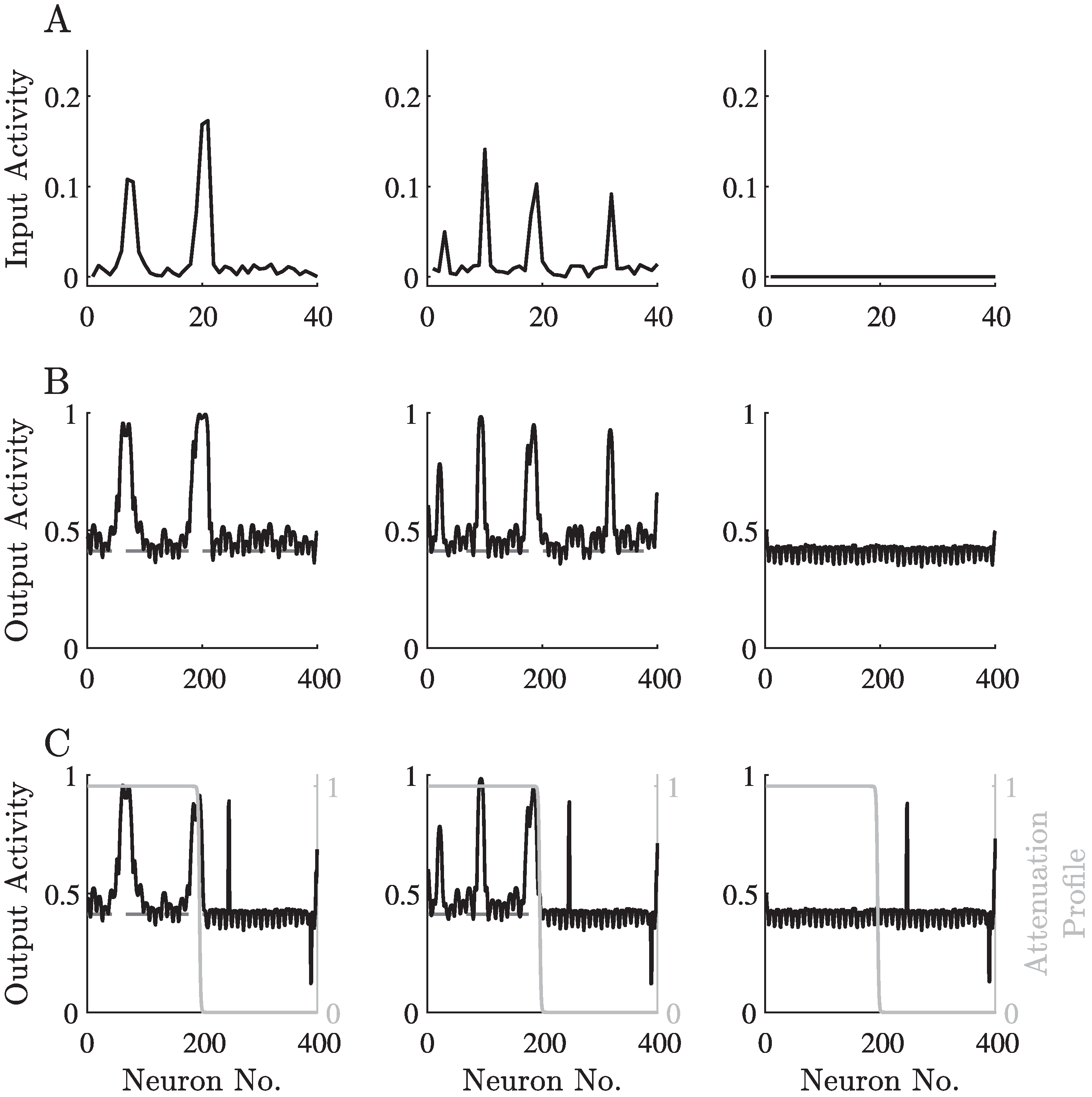

Supplement: S6 Fig — A: Typical stimuli and a silent stimulus (zero input—right panel). B: The network’s response to the stimuli presented in A. C: The network’s response to the stimuli presented in A after training on stimuli with attenuated high frequencies. The attenuation profile is depicted in gray. The spontaneous activity of the output neurons, defined here as the average activity in response to a silent stimulus before attenuation (as in the right panel of B), is indicated in B–C by a dashed line. See Fig 4 for further details. (TIF) [file pcbi.1008664.s006.tif]

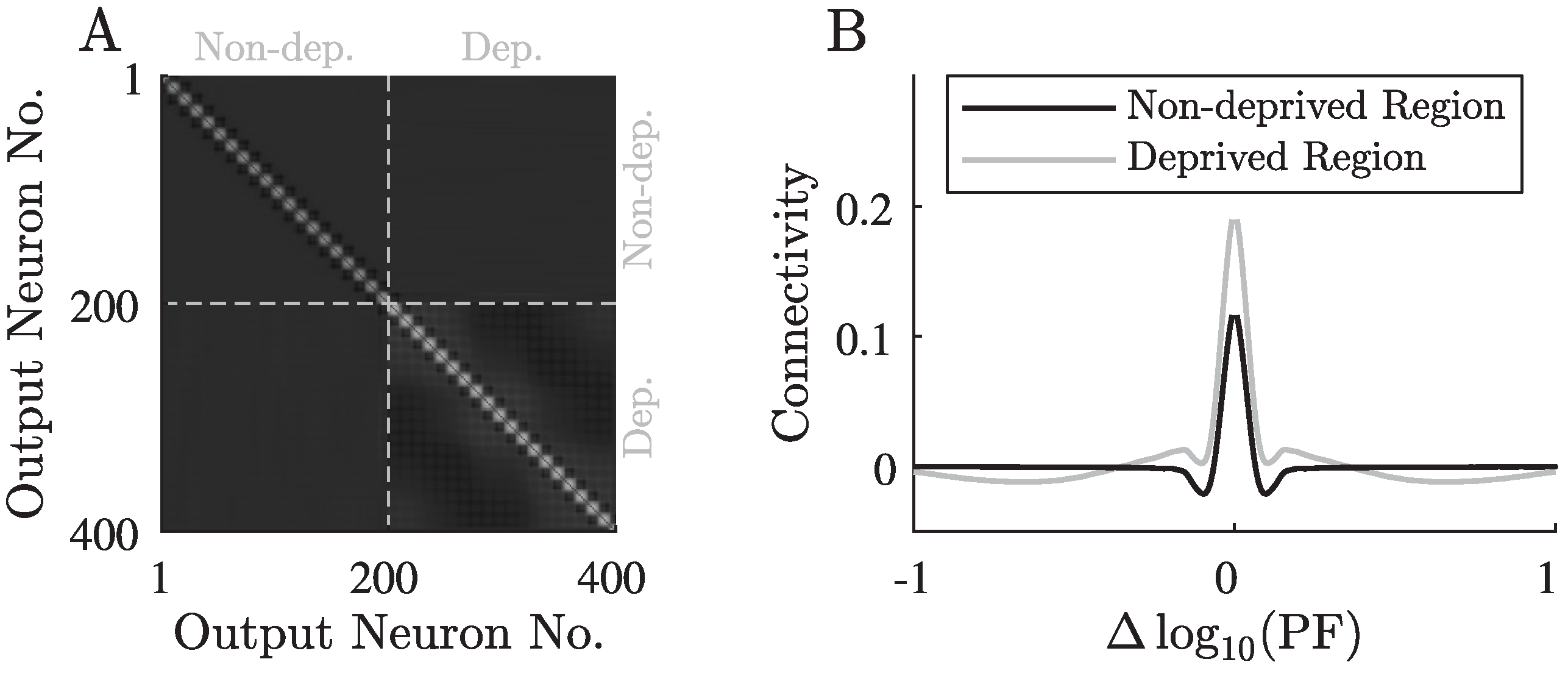

Supplement: S7 Fig — A: The recurrent connectivity matrix. B: The average row profile of the recurrent connectivity matrix, averaged separately for neurons in the deprived zone and the non-deprived zone. The attenuation profile’s parameters were k0 = 20, β = 10 (see Fig 2B). See Fig 3 for further details. (TIF) [file pcbi.1008664.s007.tif]

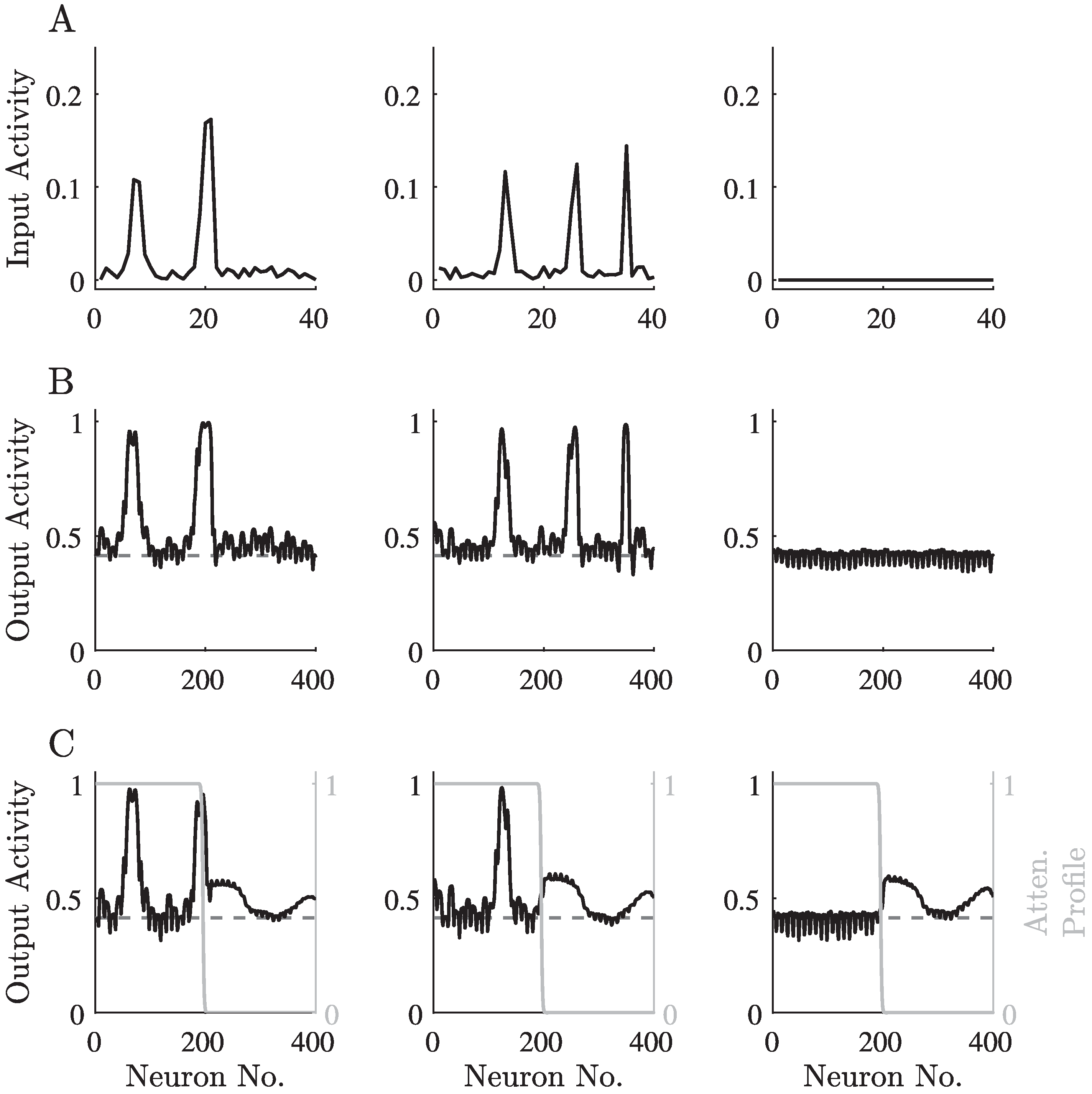

Supplement: S8 Fig — A: Typical stimuli and a silent stimulus (zero input—right panel). B: The network’s response to the stimuli presented in A after training only the feed-forward connections. C: The network’s response to the stimuli presented in A after training on stimuli with attenuated high frequencies. The attenuation profile is depicted in gray. The spontaneous activity of the output neurons, defined here as the average activity in response to a silent stimulus before attenuation (as in the right panel of B), is indicated in B–C by a dashed line. See Fig 4 for further details. (TIF) [file pcbi.1008664.s008.tif]

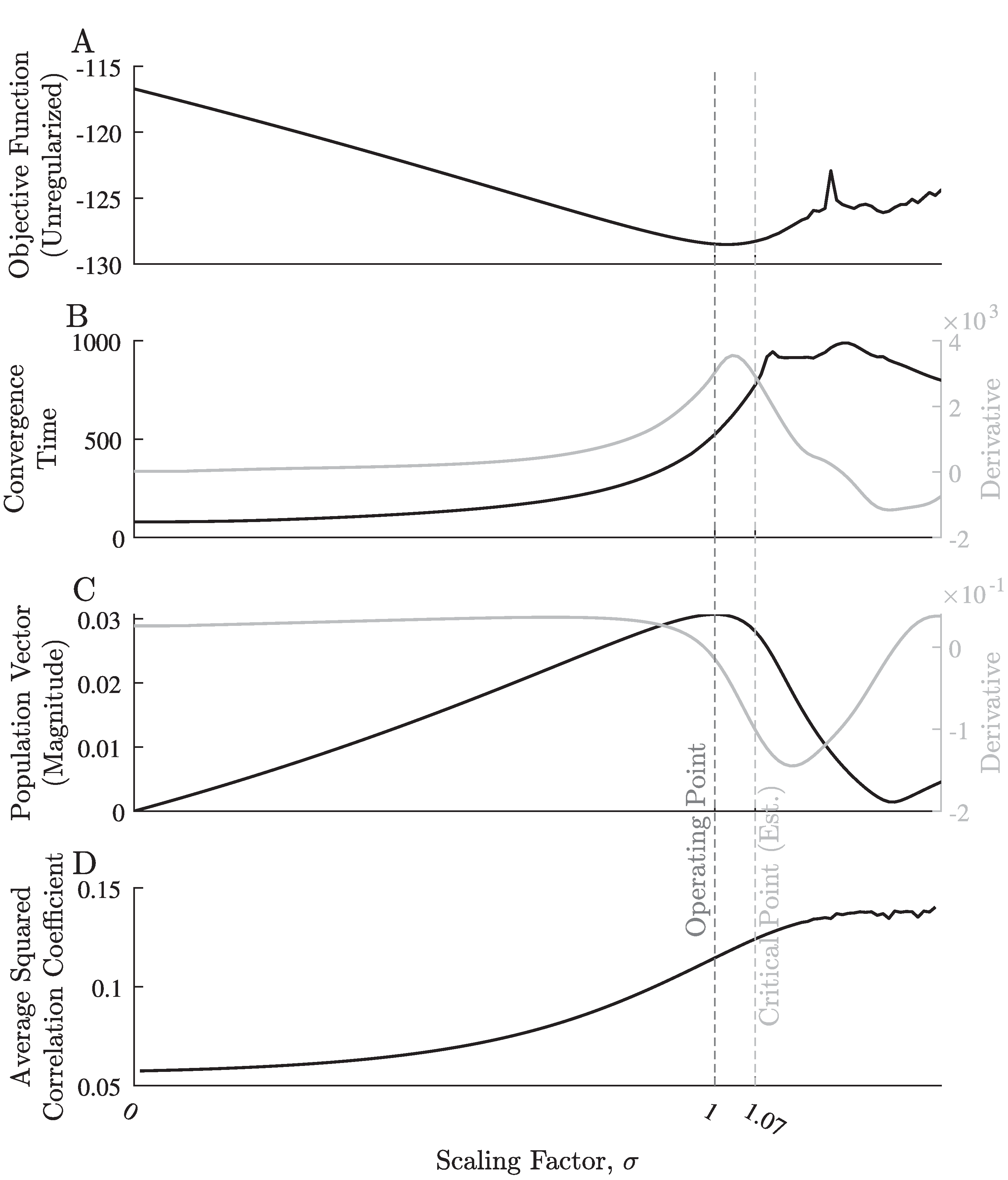

Supplement: S9 Fig — A: The network’s objective function, without the regularization terms. B: The convergence time of the network dynamics using Euler’s method. C: The population vector magnitude. D: The squared correlation coefficient between pairs of output neurons, averaged over all such pairs. All the above measures are displayed for different scaling factors of the recurrent connectivity matrix Ktr, as found by the training process; i.e., for each value of the scaling factor σ, the different measures were evaluated by replacing the recurrent connectivity matrix with K = σKtr. The recurrent connectivity matrix used here was obtained after sensory deprivation. The attenuation profile used had the parameters k0 = 20, β = 10. The operating point is at a scaling factor of 1, namely, the recurrent connectivity the learning process has converged to. The marked critical point is the scaling factor for which the spectral radius ρ(K) of the recurrent connectivity matrix is 4, i.e., 4/ρ(Ktr). See Fig 5 for further details. (TIF) [file pcbi.1008664.s009.tif]

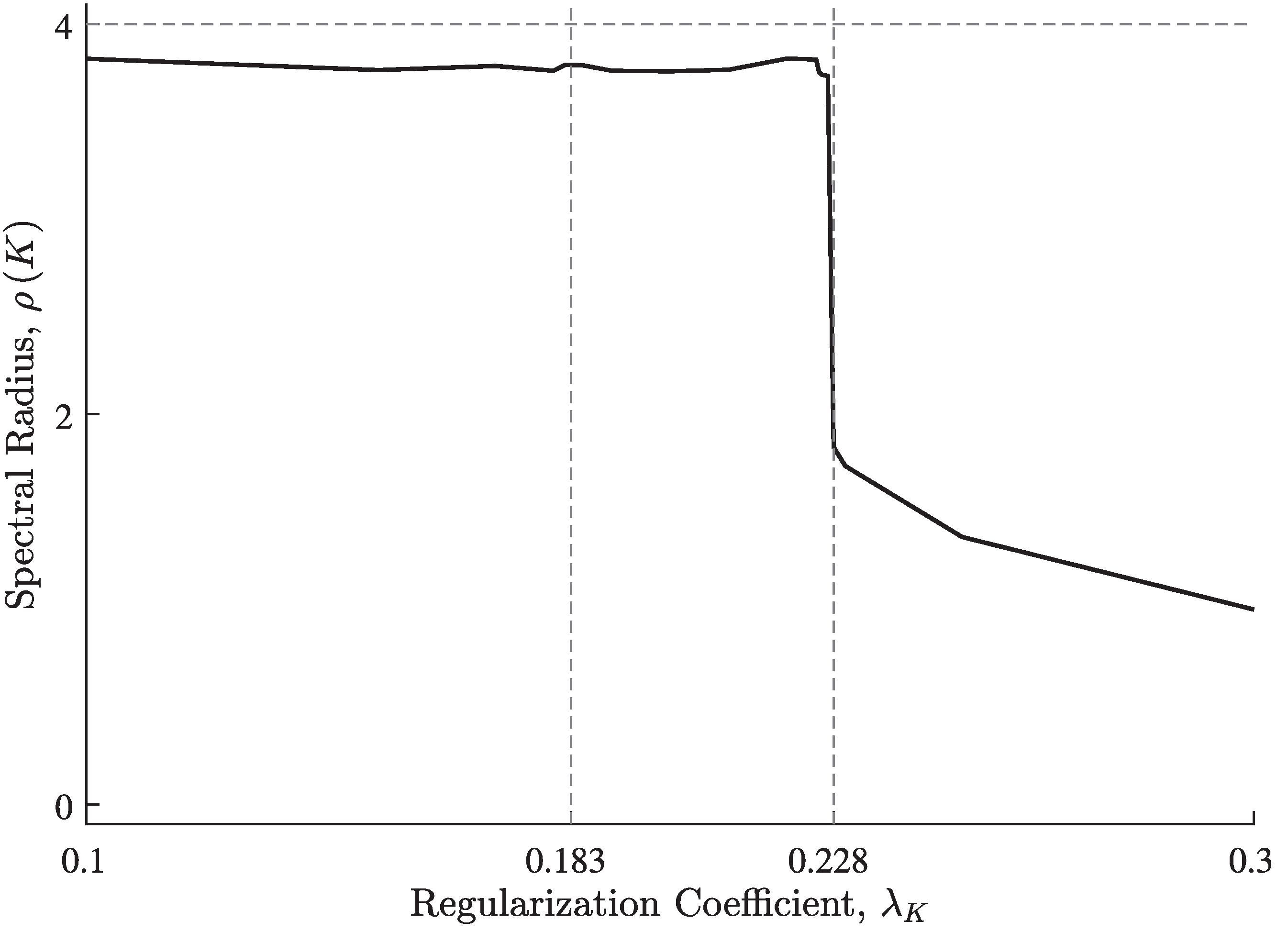

Supplement: S10 Fig — The spectral radius, ρ(K), of the recurrent connectivity matrix K as a function of the regularization coefficient λK, after the induction of sensory deprivation. See Fig 6 for further details. (TIF) [file pcbi.1008664.s010.tif]

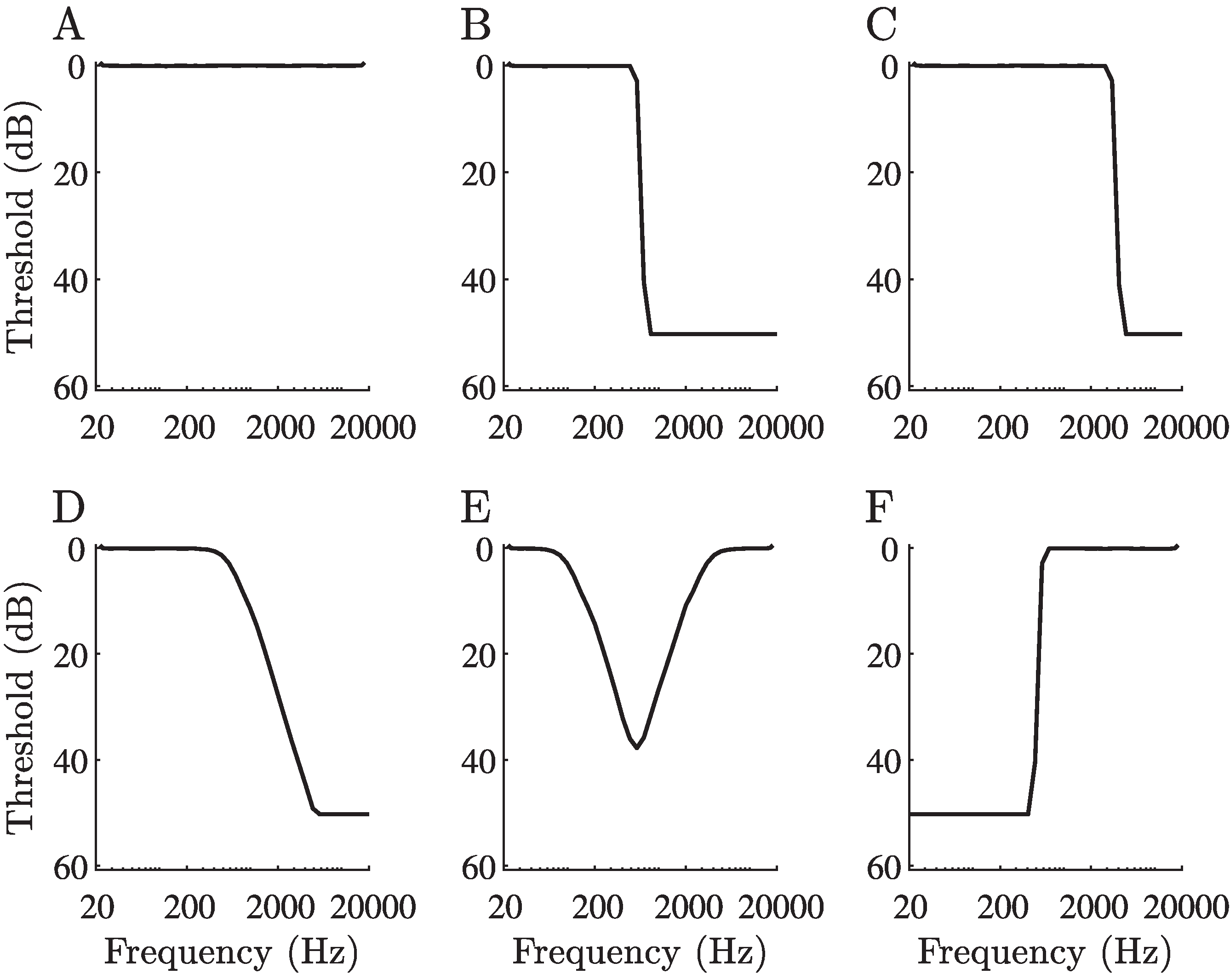

Supplement: S11 Fig — A: A simulated audiogram without sensory deprivation. B–F: Simulated audiograms for different attenuation profiles, matching the ones in Fig 2B–2F, respectively. To simulate subjective hearing thresholds, the threshold of each frequency represents the input activity required to produce a difference of 0.01 (measured by ℓ∞-norm) between a silent input and an input where only the specific frequency is active. The thresholds were found using the bisection method in the interval [0, 100], with a tolerance of 10−6. (TIF) [file pcbi.1008664.s011.tif]
